# CD8^+^ T cells retain protective functions despite sustained inhibitory receptor expression during Epstein-Barr virus infection in vivo

**DOI:** 10.1371/journal.ppat.1007748

**Published:** 2019-05-30

**Authors:** Bithi Chatterjee, Yun Deng, Angelika Holler, Nicolas Nunez, Tarik Azzi, Liliana Danusia Vanoaica, Anne Müller, Hana Zdimerova, Olga Antsiferova, Andrea Zbinden, Riccarda Capaul, Johannes H. Dreyer, David Nadal, Burkhard Becher, Mark D. Robinson, Hans Stauss, Christian Münz

**Affiliations:** 1 Viral Immunobiology, Institute of Experimental Immunology, University of Zurich, Switzerland; 2 Institute of Immunity and Transplantation, Royal Free Campus, University College London, United Kingdom; 3 Inflammation Research, Institute of Experimental Immunology, University of Zurich, Switzerland; 4 Division of Infectious Diseases and Hospital Epidemiology, Children’s Research Center, University Children’s Hospital Zurich, Switzerland; 5 Institute of Medical Virology, University of Zurich, Switzerland; 6 Institute for Pathology, Unfallkrankenhaus Berlin, Berlin, Germany; 7 Institute of Molecular Life Sciences, University of Zurich, Zurich, Switzerland; 8 SIB Swiss Institute of Bioinformatics, Zurich, Switzerland; University of California Berkeley, UNITED STATES

## Abstract

Epstein Barr virus (EBV) is one of the most ubiquitous human pathogens in the world, persistently infecting more than 90% of the adult human population. It drives some of the strongest human CD8^+^ T cell responses, which can be observed during symptomatic primary infection known as infectious mononucleosis (IM). Despite high viral loads and prolonged CD8^+^ T cell stimulation during IM, EBV enters latency and is under lifelong immune control in most individuals that experience this disease. We investigated whether changes in T cell function, as frequently characterized by PD-1 up-regulation, occur during IM due to the prolonged exposure to high antigen levels. We readily detected the expansion of PD-1 positive CD8^+^ T cells together with high frequencies of Tim-3, 2B4, and KLRG1 expression during IM and in mice with reconstituted human immune system components (huNSG mice) that had been infected with a high dose of EBV. These PD-1 positive CD8^+^ T cells, however, retained proliferation, cytokine production, and cytotoxic abilities. Multiple subsets of CD8^+^ T cells expanded during EBV infection, including PD-1^+^Tim-3^+^KLRG1^+^ cells that express CXCR5 and TCF-1 germinal center homing and memory markers, and may also contain BATF3. Moreover, blocking the PD-1 axis compromised EBV specific immune control and resulted in virus-associated lymphomagenesis. Finally, PD-1^+^, Tim-3^+^, and KLRG1^+^ CD8^+^ T cell expansion coincided with declining viral loads during low dose EBV infection. These findings suggest that EBV infection primes PD-1 positive CD8^+^ T cell populations that rely on this receptor axis for the efficient immune control of this ubiquitous human tumor virus.

## Author summary

Since its discovery as a tumor virus by Epstein and colleagues in 1964, Epstein-Barr virus (EBV) has been implicated in many serious diseases, including infectious mononucleosis, Burkitt’s lymphoma, and post-transplant lymphoproliferative disease. Currently, in vivo studies are lacking to understand the comprehensive immune control of EBV in most healthy virus carriers, and, in particular, the characteristics of the CD8^+^ T cells involved in this process. We find that even though CD8^+^ T cells express multiple inhibitory receptors including PD-1 during primary EBV infection, they appear to retain an ability to produce cytokines, to kill infected cells, and to proliferate. Importantly, blocking the PD-1 pathway leads to defects in EBV-specific control and increased virus-induced tumor formation, indicating that this axis is important for viral control. This is in contrast to previous studies where releasing an inhibitory block is important for reinvigorating immune responses against cancer. Because PD-1 function is required to keep EBV in check, this study provides evidence against blocking co-inhibitory pathways in disease settings that require improved immune control of chronic virus infections.

## Introduction

Epstein-Barr virus (EBV) is a γ-herpes virus that persists in more than 90% of the world’s adult human population. Primary EBV infection mainly occurs asymptomatically in children. However, people in the Western world often contract EBV as a primary infection in their second decade of life or later, which can then lead to infectious mononucleosis (IM) [1]. Patients with IM have fever, swollen lymph nodes, and large numbers of CD8^+^ T cell lymphoblasts. Adults with a history of IM have a significantly increased incidence of Hodgkin’s lymphoma and multiple sclerosis [2–4]. EBV^+^ individuals are at increased risk to develop any number of EBV-associated malignancies, such as nasopharyngeal carcinoma, T cell lymphomas, or Burkitt’s lymphoma [5–7]. Immunocompromised individuals are at higher risk to develop certain EBV-associated conditions, such as post-transplantation lymphoproliferative disease (PTLD) [7]. Therefore, both insufficient and hyperactive EBV specific immune responses lead to virus associated pathologies such as lymphoproliferation and IM, respectively.

EBV displays a preferential tropism for human cells, and models of primary infection have been established on the basis of where the virus can be found in healthy EBV carriers [8, 9]. EBV transforms mucosal B cells after transmission via saliva [10] and is able to maintain lifelong latent infection when some fraction of these B cells enters the memory pool as resting cells. The periodic reactivation of these latent cells may drive low level viral shedding seen in carriers [11, 12]. As with many γ-herpes viruses, EBV expresses unique proteins in its lytic and latent stages; more than eighty gene products during lytic EBV infection (including BZLF1 and BMLF1) and up to eight latent EBV antigens (EBNA1, EBNA2, EBNA3A-C, EBNA-LP, LMP1, and LMP2) are expressed depending on the differentiation stage of the infected B cell [7]. These latent EBV gene expression programs are exactly the same as those found in EBV associated malignancies [13]. Thus, immune control is thought to prevent the transition of these latency programs into lymphomagenesis.

There is evidence that CD8^+^ T cells play an important role in keeping EBV infection well-controlled in healthy individuals. CD8^+^ T cells significantly expand in acute IM [14]. While overall T cell numbers drop to normal levels over time, a significant proportion of memory CD8^+^ T cells specific for EBV is retained and increases with age, but decreases in functionality [15]. One therapy that has shown some success in EBV-driven PTLD is the introduction of expanded EBV-specific CD8^+^ T cells [16, 17]. Moreover, depletion of CD8^+^ T cells results in increased viral loads and tumor incidence with IM-like EBV infection in mice with reconstituted human immune system components (huNSG mice) [18, 19]. Further evidence for the importance of T cells in controlling EBV infection can be found in patients with certain primary immunodeficiencies. Individuals with mutations in perforin or in proteins involved in vesicular fusion for cytotoxic granule release present with uncontrolled EBV infection [20, 21]. Furthermore, primary immunodeficiencies in genes implicated in T and NK cell function such as CD27, SH2D1A, and others result in a failure to control EBV induced lymphomas, underscoring the critical need for the cytotoxic arm of the immune system to control this tumorigenic virus [22, 23]. Therefore, a detailed investigation of the CD8^+^ T cell response and function during EBV infection is necessary for understanding the pathogenesis of EBV and EBV-related diseases, and to elucidate mechanisms that could therapeutically control not only viral infection but also in general tumors that require cytotoxic lymphocytes for their rejection.

A previous study implicated a loss of EBV control during lupus to exhausted PD-1 expressing EBV-specific T cells [24]. CD8^+^ T cell exhaustion is a stepwise, dysfunctional transformation in response to persistent T cell activation caused by high antigen load and/or impaired bystander cell help, among other possible factors [25–27]. This condition leads to poor immune control of infections and tumors. T cells first lose their cytotoxic capabilities. Inhibitory and terminal differentiation receptors on their cell surface are progressively upregulated, including PD-1, Tim-3, and Lag-3, and correlate with dysfunction [28, 29]. Eventually, exhausted T cells become unable to produce IFNγ and are deleted from the repertoire [25, 26]. T cell exhaustion has mainly been described in several mouse disease models, particularly in lymphocytic choriomeningitis virus (LCMV) infection, which has a particularly high and prolonged antigen burden after infection with LCMV clone 13, and where T cell exhaustion was first described [30]. It has also been demonstrated in other chronic viral infections, including those with human immunodeficiency virus (HIV) [31, 32], hepatitis B virus [33], and hepatitis C virus [34]. It is, however, becoming increasingly clear that PD-1 expression marks a continuum of CD8^+^ T cells, encompassing recently activated effector cells to terminally differentiated stages, whose function can be partially rescued by PD-1 blockade, but which also requires PD-1 for their persistence [35–37].

While PD-1 blockade improves LCMV specific immune control by CD8^+^ T cells during acute infection [38], we demonstrate that PD-1 positive CD8^+^ T cell populations retain functionality and proliferative capacity during primary symptomatic EBV infection. We examined CD8^+^ T cells in patients with active IM, as well as in huNSG mice, which is a well-characterized in vivo model for EBV infection. We found that an increase in the frequency of inhibitory and differentiation molecules on the surface of CD8^+^ T cells, including PD-1, Tim-3, Lag-3, BTLA, 2B4, and KLRG1, was associated with elevated EBV loads. Despite the expression of PD-1, cells were able to produce cytokines associated with cytotoxicity, were positive for degranulation markers such as CD107a, and maintained proliferation. Additionally, we found that the PD-1^+^ CD8^+^ T cell population was heterogeneous in nature after EBV infection, including expanded PD-1^+^ cells that retain protective T cell functions. Importantly, PD-1 signaling seems to be required to sustain the protection by these T cell populations, because treatment with an anti-PD-1 antibody resulted in higher viral loads and elevated tumor burden in infected animals. These data together indicate that PD-1 marks functional CD8^+^ T cells during primary symptomatic EBV infection and that this is required for their protective function against this common human tumorigenic virus.

## Results

### CD8^+^ T cells express multiple inhibitory and differentiation markers during symptomatic primary EBV infection

**Fig 1:**
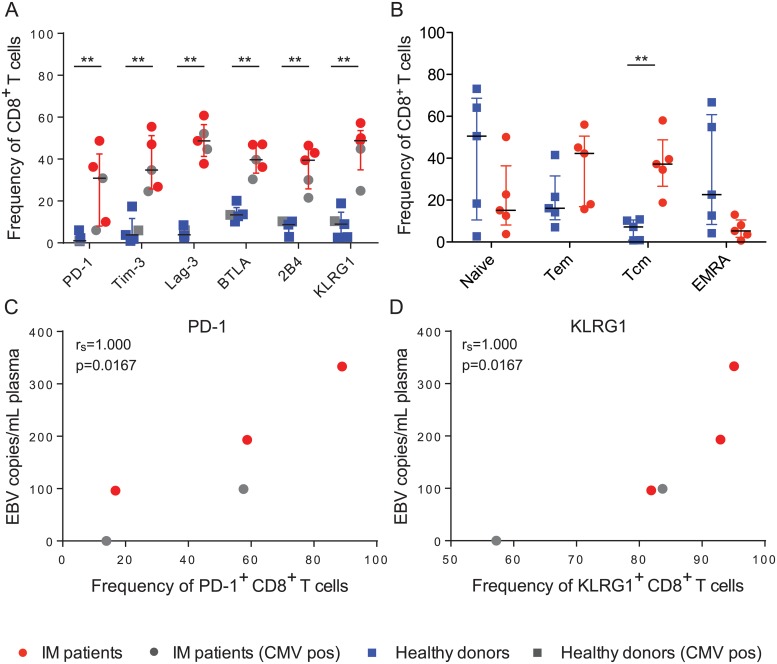
IM patients upregulate inhibitory and differentiation markers in response to EBV infection. **A)** IM and healthy donor PBMCs were antibody labeled for surface inhibitory and differentiation molecules, and analyzed by flow cytometry. All p values were 0.0079, according to the nonparametric Mann-Whitney U test, which compares distributions between two groups. Gray points indicate CMV seropositive donors. CD8^+^ T cell frequencies are displayed relative to CD45^+^ cells. **B)** Comparison of the different memory populations between healthy donors and IM patients was performed using the Mann-Whitney U test (p = 0.0079). **C)** PD-1^+^ CD8^+^ T cell frequencies were plotted relative to IM patient viral loads and analyzed using the nonparametric Spearman correlation, which examines rank correlation. **D)** As in C), but for KLRG1. For A-D, each point represents one donor; five IM patients and five healthy donors were compared. The data in A and B are displayed using the median and interquartile range. *, p<0.05 **, p<0.01.

We first examined pediatric patients that were identified based on typical symptoms and a serology compatible with infectious mononucleosis (IM), a serious, acute form of primary EBV infection. We isolated PBMCs from these individuals and together with healthy donors, examined the frequency of cells expressing receptors that have been implicated in CD8^+^ T cell function [39]. The median ages of our donor cohorts were matched, at around 10 years of age (S1A Fig). We found that multiple inhibitory receptors and differentiation markers were expressed on the surface of CD8^+^ T cells from IM patients, including PD-1, Tim-3, Lag-3, BTLA, 2B4, and KLRG1 (Fig 1A). Two of the five IM patients were seropositive for human cytomegalovirus (HCMV), but this did not correlate with higher PD-1 and KLRG1 expression on their expanded CD8^+^ T cells (gray points, Fig 1A). Surprisingly, when examining the differentiation status of these cells, it appeared that IM patients carried elevated frequencies of CD8^+^ T cells with a central memory phenotype (CD62L^+^CD45RA^-^), as compared to healthy donors (Fig 1B), which has been recently reported for influenza A virus specific CD8^+^ T cells [35]. Interestingly, we observed that viral loads positively correlated with the frequency of some receptors, including PD-1 (Fig 1C) and KLRG1 (Fig 1D). In contrast, we only found weak correlations with Tim-3 and 2B4 (S1B and S1D Fig, respectively), and no correlation with Lag-3. Thus, elevated EBV viral loads in IM patients seem to be associated with PD-1 and KLRG1 positive CD8^+^ T cells as well as with elevated frequencies of central memory T cells.

**Fig 2:**
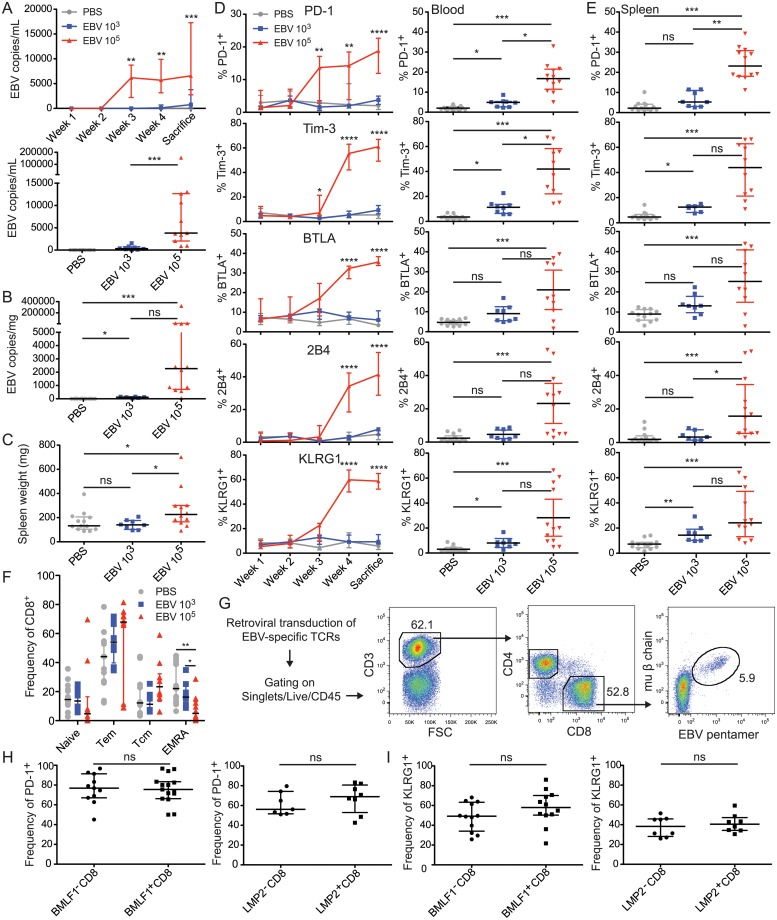
EBV infection results in higher frequencies of inhibitory and differentiation molecule expressing CD8^+^ T cells in huNSG animals. **A)** Blood viral loads are elevated upon EBV infection. (top) One representative experiment displaying longitudinal viral load development and analyzed using two-way ANOVA with Tukey’s multiple comparison test (week 3 p = 0.0073, week 4 p = 0.0034, and sacrifice p = 0.0008). (bottom) Combined experimental data were analyzed at week 5 using the Mann-Whitney U test. Because PBS values were all 0, the p value represents the comparison between the EBV 10^3^ and EBV 10^5^ groups (p = 0.0006). **B)** Spleen viral loads. p<0.0001 using the Kruskal-Wallis test. **C)** Extent of splenomegaly observed. p = 0.0305 using the Kruskal-Wallis test. **D)** (left) Blood: One representative experiment to display longitudinal increases in receptor-expressing CD8^+^ T cell frequencies, and analyzed using two-way ANOVA with Tukey’s multiple comparison test. (right) Combined data at week 5 post-infection. Kruskal-Wallis results were as follows: PD-1 p<0.0001, Tim-3 p<0.0001, BTLA p = 0.0035, 2B4 p = 0.0005, KLRG1 p<0.0001. **E)** Combined spleen data examining changes in receptor-expressing CD8^+^ frequencies. Kruskal-Wallis results were as follows: PD-1 p<0.0001, Tim-3 p<0.0001, BTLA p = 0.0033, 2B4 p = 0.0006, KLRG1 p<0.0001. **F)** Frequency of CD8^+^ as a proportion of various T cell differentiation states. Tem = effector memory T, Tcm = central memory T. Data were analyzed using the Kruskal-Wallis test (EMRA: p = 0.0052, other conditions: ns}. **G)** Gating scheme for transduced CD8^+^ T cells containing EBV-specific TCR using an antibody specific for the murine TCR beta chain and the corresponding EBV-specific pentamer. Animals were infected with EBV in this instance. **H)** EBV-specific and non-specific T cells express similar frequencies of PD-1 relative to their parent population. Lytic EBV antigen-specific CD8^+^ T cells (BMLF1, top), latent EBV antigen-specific CD8^+^ T cells (LMP2, bottom). **I)** As in H, but displaying KLRG1. The representative data from A-D comprised 4–5 animals per group. The combined data from A-F were analyzed using the Kruskal-Wallis test, and statistics displayed are the results of the Dunn’s post-test. *, p<0.05, **, p<0.01, ***, p<0.001, ns = not significant. PBS (n = 13), EBV 10^3^ (n = 8), EBV 10^5^ (n = 12). The data from H-I were analyzed using the Mann-Whitney U test. All data in Fig 2 are displayed using the median and interquartile range, and each point represents one animal. All combined data are from 4 independent experiments.

Given that EBV has a preferential tropism for human cells, studies of this virus have been limited to in vitro systems in the past. However, with advances in the generation of mice with reconstituted human immune system components (huNSG) [40–42], we were interested in examining the longitudinal acquisition of CD8^+^ T cell phenotypes and function in response to EBV infection in such an animal model. Briefly, huNSG mice with or without the HLA-A*0201 transgene were reconstituted with HLA-A2^+^ CD34^+^ hematopoietic progenitor cells (huNSG mice), as outlined in S2A Fig [43]. These animals had an average human CD45^+^ reconstitution of (65%), with around 40% CD3^+^ T cells, around 55% CD19^+^ B cells, as well as around 3% NKp46^+^ NK cells (S2A Fig). The T cell compartment was comprised of approximately 25% CD8^+^ T cells and 75% CD4^+^ T cells (S2A Fig). Animals were infected with EBV and were followed for a five-week period (S2B Fig). We observed that infected huNSG mice exhibited blood viral loads that became evident at week 3 post-infection for a higher EBV dose of 10^5^ infectious particles, and at week 5 for a lower dose of EBV with 10^3^ infectious particles (Fig 2A, top). Viral loads in high EBV dose-infected animals were significantly elevated at week 5 in the blood (Fig 2A, bottom), as well as in the spleen (Fig 2B). High dose infected animals exhibited signs of splenomegaly (Fig 2C), much like IM patients [44]. Therefore, taken together, high dose EBV infection can elicit an IM-like primary infection in huNSG mice.

We examined the CD8^+^ T cell compartment longitudinally in the blood of EBV infected huNSG mice (Fig 2D, left). We observed a striking and sustained increase in the frequency of cells that are positive for PD-1, Tim-3, BTLA, 2B4, and KLRG1, approaching similar frequencies to what we observed in IM patient samples. This increase appeared to track closely with the increase in blood viral loads (Fig 2A). The differences observed between the higher and lower dose of EBV infection together with PBS controls were significant (Fig 2D, right). We observed similar trends in receptor frequencies in the spleen (Fig 2E). These data were confirmed using tSNE analysis, where we observed that these receptors were expressed mainly by the T cell compartment (S3A and S3B Fig). During this analysis, we observed an inverse relationship between the expression of PD-1, Tim-3, or 2B4 and the expression of CD127 (IL-7R) on T cells. We further examined whether EBV infection affects the differentiation state of CD8^+^ T cells. Based on the frequencies of cells expressing CD62L and/or CD45RA, we observed a tendency towards an increase in the central memory population, and a significant decrease in the EMRA population (Fig 2F). These data indicate that the CD8^+^ T cells that arise during primary EBV infection in huNSG mice express many receptors implicated in inhibitory processes known for poor disease control.

In order to assess whether these observations also extended to EBV-specific CD8^+^ T cells, we examined the behavior of BMLF1 (early lytic EBV antigen) and LMP2 (latent EBV antigen)-specific T cells introduced into huNSG mice prior to infection. For this purpose, splenocytes were retrovirally transduced with recombinant T cell receptors (TCRs) specific for the immunodominant HLA-A2-restricted BMLF1 aa259-267 (GLCTLVAML) or LMP2 aa426-434 (CLGGLLTMV) peptides and adoptively transferred into donor-mate huNSG mice (S4A Fig), which were then EBV infected. These cells were able to produce IFNγ, TNFα, and IL-2 in response to their cognate peptides after transduction (S4B Fig). We were able to track these cells specifically during our in vivo infection experiments due to the presence of a murine constant region in the TCR beta chain in the construct [45]. The transduced cells of interest were human EBV peptide/HLA-A2 pentamer^+^ and murine Cβ chain^+^ (Fig 2G). We observed that the frequency of PD-1 expressing lytic or latent EBV-specific cells appeared to track closely with that observed in the overall T cell population (Fig 2H). Similar frequencies of KLRG1 were also observed when examining these EBV-specific T cell populations (Fig 2I). These data indicate that EBV-specific T cells appear to be phenotypically similar to the overall T cell population that expands during EBV infection in huNSG mice.

### IM patients and huNSG mice infected with EBV retain unique transcriptional characteristics

**Fig 3:**
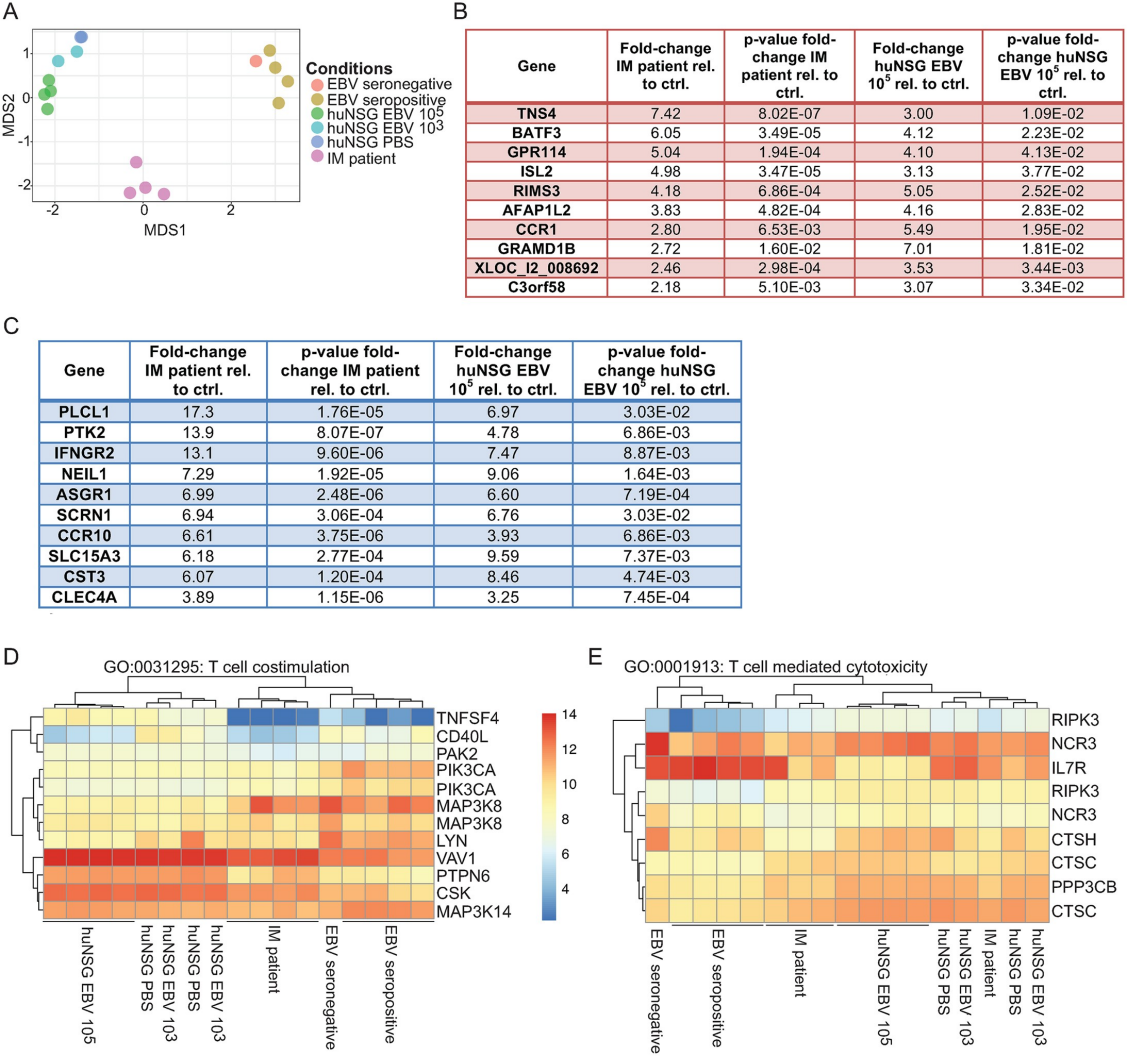
IM patients and huNSG mice infected with EBV retain unique transcriptional characteristics. **A)** The data generated from gene microarray was subject to multidimensional scaling. Samples that are more similar cluster together. Each point corresponds to one donor or animal. **B)** The top ten genes that are upregulated in both IM patients and high dose huNSG mouse infection, together with the p values indicating significance of fold-change. **C)** The top ten genes that are downregulated in both IM patients and high dose huNSG mouse infection, together with the p values indicating significance of fold-change. **D)** Selected genes (right) from the GO term T cell costimulation and their up (red) or downregulation (blue) across patient or huNSG mouse conditions (bottom). **E)** Selected genes (right) from the GO term T cell mediated cytotoxicity and their up (red) or downregulation (blue) across patient or huNSG mouse conditions (bottom).

The similarities in inhibitory receptor up-regulation and central memory CD8^+^ T cell expansions during high dose EBV infection of huNSG mice and IM prompted us to take a more unbiased look at the similarities between CD8^+^ T cells that expand in these two symptomatic primary EBV infection conditions. For this purpose, we purified CD8^+^ T cells from IM patients (n = 4), healthy EBV carriers (n = 4), and one EBV seronegative individual, as well as from high EBV dose (n = 4), low dose (n = 2), and uninfected (n = 2) huNSG mice. The RNA of these preparations was subjected to gene microarray and multidimensional scaling (MDS) (Fig 3A). The transcriptomes of EBV negative samples clustered with those of healthy EBV carriers, and low EBV dose infected huNSG mice clustered separately due to species origin. While the species origin produced the most significant difference in MDS1, MDS2 distinguished high dose from low dose or uninfected samples as well as IM samples from healthy EBV carriers and seronegative samples (Fig 3A). When comparing the transcripts of IM patients and high dose huNSG relative to their controls, the most prominently co-up-regulated transcripts include BATF3 (Fig 3B), a transcription factor that was previously primarily implicated in the biology of antigen cross-presenting dendritic cells [46], but which has been also found to be up-regulated in memory CD8^+^ T cells in gene microarray analysis [47]. In the list of down-regulated genes, the β chain of the IFNγ receptor is prominently co-down-regulated (Fig 3C). Since these main co-regulated genes did not give us any additional functional insights into co-stimulation and protective effector functions during EBV infection, we focused our attention toward the gene ontology (GO) terms of T cell co-stimulation and T cell mediated cytotoxicity (Fig 3D and 3E, S5 Fig). We observed that OX40L and CD40L were transcriptionally down-regulated during symptomatic primary EBV infection in huNSG mice and IM patients (Fig 3D), both of which had been observed to be up-regulated in RNA sequencing datasets during prolonged LCMV infection [47]. Within the cytotoxicity GO term, we observed CD127 (IL-7R) down-regulation (Fig 3E), which confirmed that EBV-driven CD8^+^ T cell expansion leads to PD-1, Tim-3, or 2B4 inhibitory receptor expressing cell populations that our tSNE analysis already revealed to be mostly IL-7R negative (S3 Fig). Therefore, the gene expression analysis did not reveal significant additional co-stimulatory receptors that are up-regulated on the CD8^+^ T cell subsets that expand during IM and high dose EBV infection of huNSG mice.

### CD8^+^ T cells retain proliferative capacity during prolonged symptomatic EBV infection in vivo

**Fig 4:**
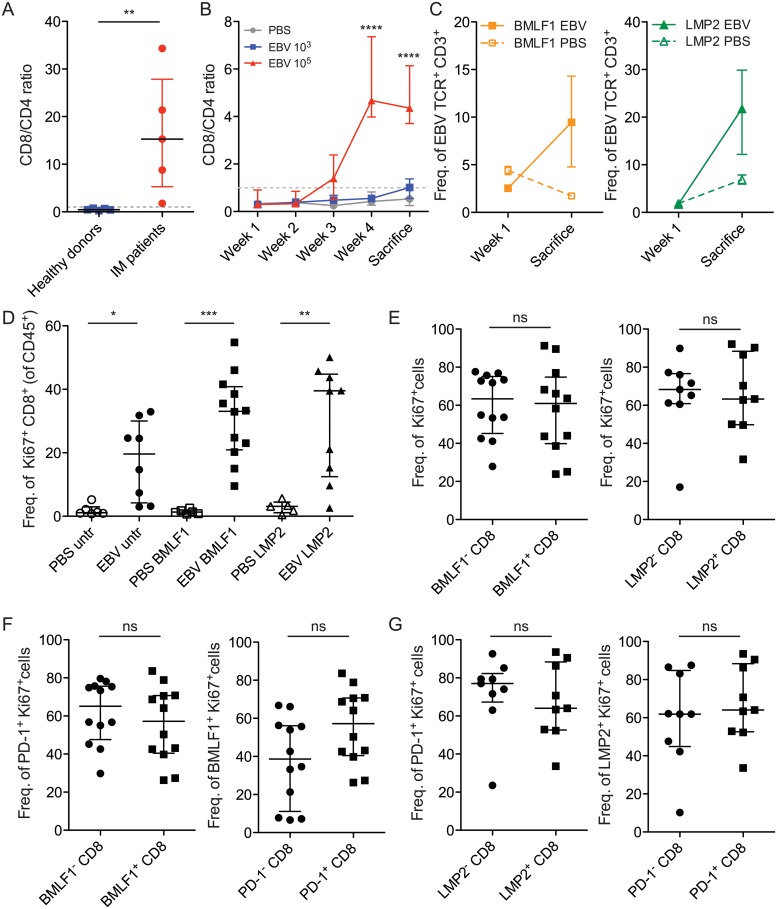
CD8^+^ T cells retain proliferative capacity during prolonged symptomatic EBV infection in vivo. **A)** CD8/CD4 ratio comparing CD8^+^ T cell expansion in IM patient PBMC samples with healthy donors. Each dot represents one donor (IM patients: n = 5, healthy donors: n = 5). Data were analyzed using the non-parametric Mann-Whitney U test, p = 0.0079. **B)** Longitudinal changes in overall CD8/CD4 ratio from one representative huNSG experiment of 5 (n = 4–5 animals/group). ****, p<0.0001, two-way ANOVA with Tukey’s multiple comparison test. **C)** Frequency of CD3 expressing transduced EBV TCRs as outlined in legend, upon 10^5^ EBV infection (EBV) or PBS treatment, in one representative experiment of 4, with 2–5 animals per group. **D)** Data examining the frequency of Ki67^+^ CD8^+^ cells relative to CD45^+^ in huNSG animals transferred with untransduced cells (untr), BMLF1^+^ cells (BMLF1) or LMP2^+^ cells (LMP2), and treated with either PBS or EBV 10^5^ (EBV). Data were compared using the Kruskal-Wallis test (p<0.0001) and further analyzed using a Dunn’s post-test. *, p<0.05, **, p<0.01, ***, p<0.001. **E)** Frequency of Ki67^+^ CD8^+^ cells in EBV-TCR transduced populations compared to non-transduced populations. **F)** As in E), but examining the frequency of PD-1^+^ Ki67^+^ CD8^+^ cells; (right) Examining the frequency of BMLF1^+^ Ki67^+^ CD8^+^ cells relative to PD-1 CD8. The same data set is represented in both graphs. G) (left) As in E), but examining the frequency of PD-1^+^ Ki67^+^ CD8^+^ cells relative to LMP2 CD8; (right) Examining the frequency of LMP2^+^ Ki67^+^ CD8^+^ cells relative to PD-1 CD8. The same data set is represented in both graphs. For D-G, data displayed were combined from 3 independent experiments. E-G were analyzed using the Mann-Whitney U test. ns = not significant. Data from all figures are displayed as the median and interquartile range, and each point represents one animal *, p<0.05, **, p<0.01, ***, p<0.001, ns = not significant.

CD8^+^ T cells are known to greatly expand during acute symptomatic EBV infection [48]. We were interested in examining whether cells retained their proliferative capacity during EBV infection, because exhausted PD-1^+^ T cells have been shown to lose their expansion potential [49]. We examined the CD8/CD4 ratio of IM patients as a measure of CD8^+^ T cell expansion. Compared to healthy donors that generally had a CD8/CD4 ratio of less than one (depicted by dashed gray line), IM patients had significantly higher frequencies of CD8^+^ T cells (Fig 4A). We followed the expansion of CD8^+^ T cells in response to EBV infection in huNSG mice (Fig 4B). With the IM-like dose of infection (10^5^ infectious EBV particles), we observed CD8^+^ T cell expansion concomitant with the appearance of viral loads (Fig 1A). With a lower dose of EBV (10^3^ infectious particles), CD8^+^ T cells appeared to expand in a delayed fashion. When we introduced lytic (BMLF1) or latent (LMP2) EBV antigen specificities as TCR transgenic CD8^+^ T cells in vivo, these cells also appeared to expand more than 3–5 fold in frequency during the course of EBV infection with 10^5^ infectious particles, compared to PBS treated controls (Fig 4C). This is comparable to the expansion rate of CD8^+^ T cells that has been observed in IM patients [50, 51]. These data indicate that CD8^+^ T cells expand similarly during symptomatic primary EBV infection in IM patients and in huNSG mice.

We investigated the active proliferative capacity of CD8^+^ T cells by examining Ki67, a protein that is expressed during all phases of the cell cycle, with the exception of the G_0_ phase. We observed that the frequency of Ki67^+^ CD8^+^ T cells increases significantly with EBV infection with 10^5^ infectious particles compared to PBS treatment, across all transferred EBV specificities (Fig 4D). Rather than observing a reduction in their proliferative capacity, this indicates that CD8^+^ T cells are capable of active cell division during infection. We observed similar relative frequencies of Ki67^+^ cells when examining BMLF1 specific CD8^+^ T cells and bulk CD8^+^ T cells (Fig 4E, left). The same was true for LMP2 specific CD8^+^ T cells (Fig 4E, right). Importantly, PD-1^+^ cells that expressed Ki67 were observed in the BMLF1 specific CD8^+^ T cell populations, and this was comparable to the corresponding BMLF1 negative population (Fig 4F, left). When examining the BMLF1 specific population, PD-1^+^ cells also tended to express Ki67 to similar or slightly higher frequencies as the PD-1 negative population, indicating that despite the presence of this inhibitory receptor, cells retained marks of active cell division (Fig 4F, right). We observed similar trends when examining LMP2 specific CD8^+^ T cell populations (Fig 4G). These data indicate that CD8^+^ T cells during EBV infection proliferate actively despite the expression of the inhibitory receptor PD-1.

### IM patients and huNSG produce proinflammatory cytokines after EBV infection

**Fig 5:**
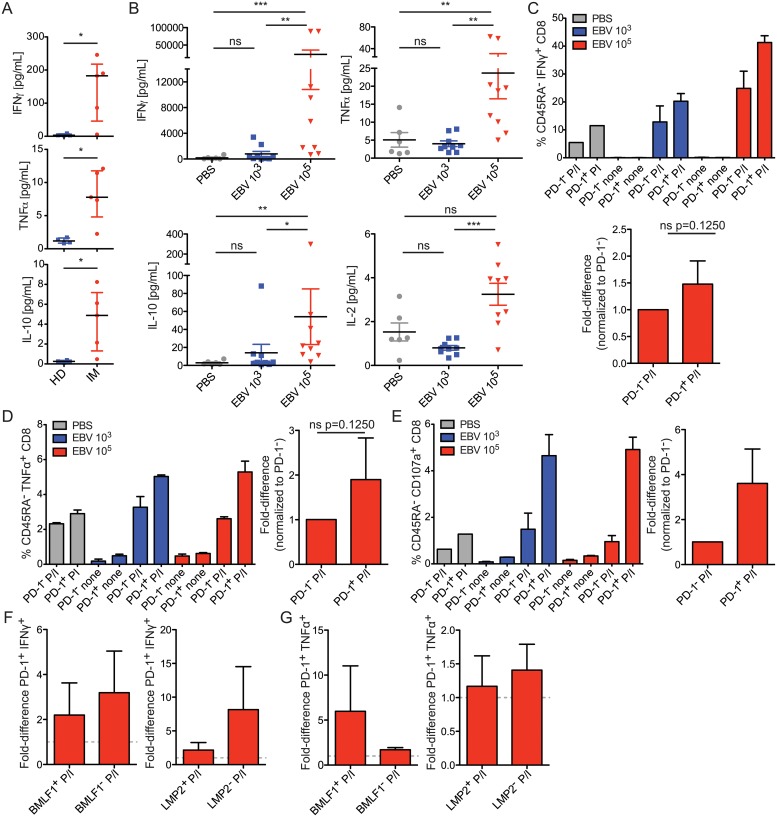
CD8^+^ T cells produce proinflammatory cytokines after EBV infection. **A)** Elevated levels of proinflammatory cytokines are found in IM patient plasma and in **B)** huNSG animal serum. In A, data were analyzed using the Mann-Whitney U test. IFNγ: p = 0.0317, TNFα: p = 0.0159, IL-10: p = 0.0159. Each point represents one donor. IM patients n = 5, healthy donors n = 4. Healthy donors used are not median-age matched to IM patients. For B, the data displayed for huNSG animals were combined from 3 independent experiments, with each point representing one animal. Data were analyzed using the Kruskal-Wallis test: IFNγ: p = 0.0015, TNFα: p = 0.0028, IL-10: 0.0042, IL-2: p = 0.0035. Results from the Dunn’s post-test are shown. **C)** CD8^+^ T cells were isolated from splenocytes, and were stimulated for up to 8 hours with or without PMA/ionomycin (P/I), and frequencies of IFNγ-expressing cells were assessed. One representative experiment of four is shown (left, displayed as mean +/- SD) and a composite of data from EBV 10^5^ P/I treated animals is shown (right). The composite 10^5^ P/I data was normalized to PD-1^-^ CD8^+^ T cells and their IFNγ expression (across 4 experiments) and analyzed using the Wilcoxon signed-rank test. **D)** As for C, examining TNFα-frequencies. One representative of four experiments is shown, and the 10^5^ P/I data are summarized and normalized to PD-1^-^ TNFα frequencies (across 4 experiments), and analyzed using the Wilcoxon signed-rank test. **E)** As for C, examining the frequency of CD107a-expressing cells. One representative of two experiments is shown (left), and a PD-1^-^ normalized composite of 2 experiments is shown to the right. **F)** P/I data normalized to PD-1^-^ comparing IFNγ frequencies in PD-1^+^ BMLF1 (left) or LMP2 (right) positive or negative populations, from 2 experiments. **G)** As in F), but examining TNFα^+^ frequencies, from 2 experiments. For all combined data sets in this figure, data are displayed with the median and interquartile range.

Because CD8^+^ T cells of EBV infected huNSG mice seem to express inhibitory receptors, but yet maintain their proliferative capacity, we hypothesized that some level of cytokine production might also remain despite persistent infection for one month with high antigenic load. We first examined the plasma of IM patients during acute infection as well as the serum of infected huNSG animals at the time of sacrifice for the presence of proinflammatory cytokines and other factors. We observed that IM patients had significantly elevated levels of IFNγ and TNFα as well as IL-10 in their plasma as compared to healthy controls (Fig 5A), while changes in IL-2 and IL-12p70 levels were either undetectable or insignificant (S6A Fig). We observed that huNSG mice that were infected with 10^5^ infectious EBV particles also had significantly higher serum levels of IFNγ, TNFα, IL-10, and IL-2 compared to infection with 10^3^ infectious EBV particles or PBS treated huNSG animals (Fig 5B). These animals exhibited other hallmarks of activation, including serum IL-13 (S6B Fig), and the presence of other chemokines and factors such as IP-10, MCP1, CCL3, CRP, and soluble adhesion molecules (S6C and S6D Fig). These data indicate that EBV infection results in inflammatory immune responses that are maintained during severe acute infection 4 to 6 weeks after EBV encounter in humans and in huNSG animals.

Surprisingly, when we gated on antigen-experienced (CD45RA^-^) IFNγ or TNFα producing CD8^+^ T cells, PD-1 expression tended to mark similar or higher production of these cytokines, especially after high dose EBV infection (Fig 5C and 5D). The responsiveness of the cells also appeared to increase with EBV dose escalation. Furthermore, degranulation (CD107a surface expression) as a surrogate of cytotoxicity, which constitutes the main protective CD8^+^ T cell function during EBV infection, appeared largely confined to PD-1 positive cell populations (Fig 5E). PD-1 expression relative to negative cells also did not compromise the cytokine production of BMLF1 and LMP2 specific CD8^+^ T cell populations (Fig 5F and 5G). Thus, PD-1 expression marks functional CD8^+^ T cells after 5 weeks of symptomatic primary EBV infection.

### PD-1^+^ CD8^+^ T cells co-express multiple inhibitory and differentiation receptors and retain functionality

**Fig 6:**
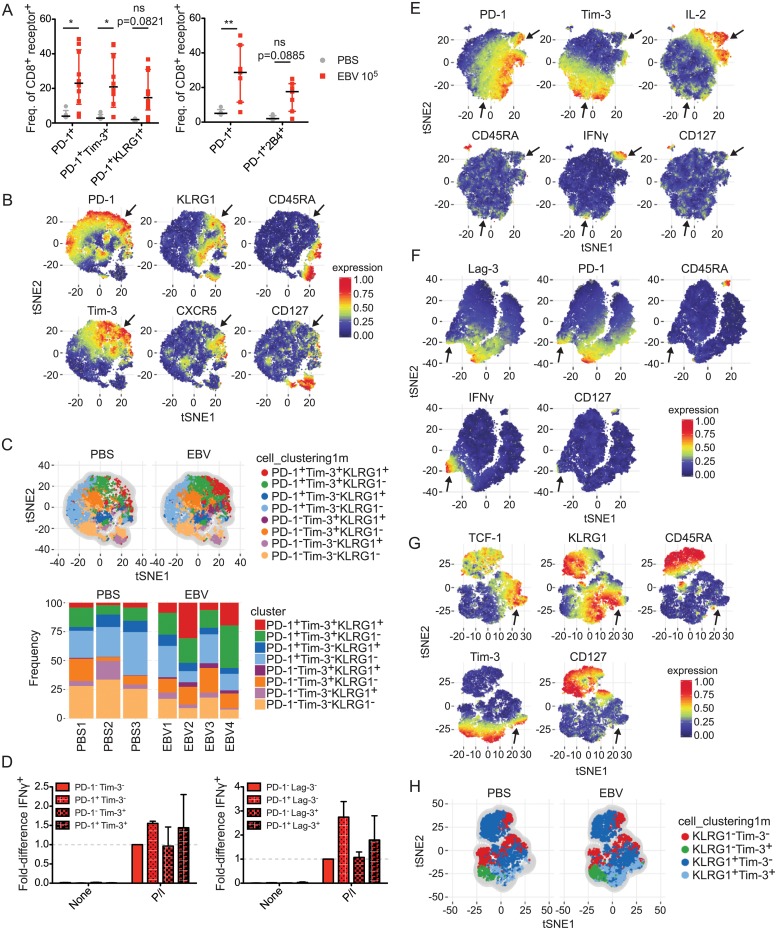
PD-1^+^ CD8^+^ T cells co-express multiple inhibitory and differentiation receptors and retain functionality. **A)** Frequency of co-expression of Tim-3 and KLRG1 (left, 3 experiments) and 2B4 (right, 2 experiments) with PD-1 with n = 5–10 animals/group. Data were analyzed using two-way ANOVA with Sidak’s post hoc test and displayed using the median and interquartile range. *, p<0.05, **, p<0.01, **B)** tSNE analysis of the CD8^+^ T cell population, where red indicates higher expression. **C)** Cell clustering analysis of the data from B), comparing PBS and high dose EBV conditions in huNSG animals and the frequencies of inhibitory and differentiation receptor containing populations in a tSNE plot (top), and graphically (bottom). **D)** Relative IFNγ expression in response to P/I across various PD-1 populations: Tim-3 (left) and Lag3 (right). Data are compiled from 2 independent experiments. Two-way ANOVA with Sidak’s post hoc test was used and data are displayed as median and interquartile range. Differences were not statistically significant between P/I groups. **E)** tSNE analysis of the CD8^+^ T cell population examining the coexpression of PD-1, Tim-3, and CD127 together with IL-2 and IFNγ. **F)** tSNE analysis of the CD8^+^ T cell population examining the coexpression of PD-1, Lag-3, and CD127 together with IFNγ. **G)** tSNE analysis of the CD8^+^ population examining the coexpression of CD127, Tim-3, and KLRG1 together with TCF1. **H)** Examining the coexpression of Tim-3 and KLRG1 relative to the data in G).

We next analyzed if the up-regulation of additional inhibitory receptors or differentiation markers would compromise this reactivity. Many PD-1 positive CD8^+^ T cells were also positive for Tim-3 and KLRG1, while around half of them also carried 2B4 after high dose EBV infection of huNSG mice (Fig 6A). This also became apparent during tSNE analysis of CD8^+^ cell populations, in which Tim-3, KLRG1, and 2B4 occupied to a large extent a similar space as PD-1 expression, but clustered away from IL-7R and CD45RA expression (Fig 6B and S7A Fig). PD-1^+^ CD8^+^ T cells were partially positive for CXCR5 and BTLA. With respect to receptor co-expressing subsets, mainly PD-1^+^Tim3^+^KLRG1^+^, PD-1^+^Tim3^+^KLRG1^-^, PD-1^+^2B4^+^BTLA^+^, and PD-1^+^2B4^+^BTLA^-^ CD8^+^ T cell populations expanded during symptomatic primary EBV infection in huNSG mice (Fig 6C and S7B Fig). Interestingly, we found a tendency for lower IFNγ production in Tim-3 or Lag-3 single-positive CD8^+^ T cells, but PD-1 co-expression seemed to rescue this reactivity to some extent (Fig 6D) upon PMA and ionomycin activation. In tSNE analysis some of the PD-1^+^Tim3^+^ CD8^+^ T cells appeared to express low levels of IL-2 in addition to IFNγ (Fig 6E, arrows), For the PD-1^+^Lag3^+^ CD8^+^ T cells we could also confirm IFNγ production in a subset of cells (Fig 6F, arrow). In addition to this, a substantial proportion of the IFNγ producing PD-1 positive CD8^+^ T cells also expressed granzyme B and degranulated (S7C Fig), suggesting that they also maintained cytotoxic function. We propose that functionality is maintained in some of the PD-1 positive CD8^+^ T cells that expand during primary EBV infection. We observed a subset of cells that did not express CD127 but co-expressed Tim-3 and KLRG1 as well as T cell factor 1 (TCF1) (Fig 6G), which has been recently shown to sustain memory function during chronic viral infections [52]. Indeed, this Tim3^+^KLRG1^+^TCF1^+^ CD8^+^ T cell population seems to expand during EBV infection (Fig 6H). Therefore, PD-1^+^ CD8^+^ T cells that co-express multiple receptors including Tim-3, KLRG1, and 2B4 expand during symptomatic primary EBV infection and appear to retain protective T cell functions like cytokine production and cytotoxicity.

### Treatment with anti-PD-1 antibodies results in higher viral loads and tumor burdens for EBV-infected animals

**Fig 7:**
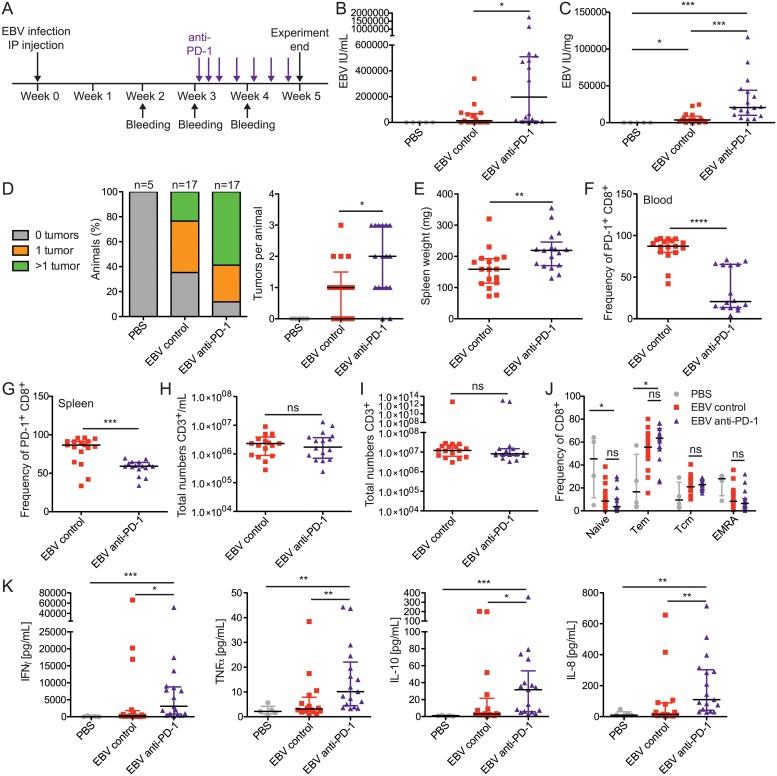
Treatment with anti-PD-1 antibodies results in higher viral loads and tumor burdens for EBV-infected animals. **A)** Schematic displaying experimental timeline. **B)** Blood viral loads across treatment groups. Animals were treated with either 100 or 150 μg of anti-PD-1 antibody per dose (EBV anti-PD-1) or with either PBS or 150 μg of isotype control antibody per dose (EBV control). Because PBS values were all 0 (n = 5), EBV control (n = 17) and EBV anti-PD-1 (n = 17) were compared using the Mann-Whitney U test (p = 0.0202). **C)** Splenic viral loads. Conditions were compared using the Kruskal-Wallis test (p<0.0001), and statistics from the Dunn’s post-test are displayed. **D)** Frequency of animals with indicated tumor numbers (left); graphical representation of tumors per animal (right). EBV control and EBV anti-PD-1 groups were compared using the Mann-Whitney U test (p = 0.0191). **E)** Extent of splenomegaly (p = 0.0065, Mann-Whitney U test). **F)** Graph indicating drop in frequency of blood PD-1^+^ cells, indicating occupancy by anti-PD-1 antibody on cell surface (p<0.0001, Mann-Whitney U test). **G)** Similar to F, but for spleen (p = 0.0005, Mann-Whitney U test). **H)** Total numbers of CD3^+^ T cells in blood and **I)** spleen, analyzed using the Mann Whitney U test. **J)** Frequency of CD8^+^ T cells after treatment as a proportion of various T cell differentiation states. Tem = effector memory T, Tcm = central memory T. Pooled data from 3 experiments were analyzed using the Kruskal-Wallis test, and statistics from the Dunn’s post-test are displayed. **K)** Serum cytokines at the time of sacrifice. Data were analyzed using the Kruskal-Wallis test (IFNγ: p = 0.0009, TNFα: p = 0.0015, IL-10: p = 0.0004, IL-8: p = 0.0013), and statistics from the Dunn’s post-test are displayed. In all panels, data displayed were combined from 3 independent experiments, with 5–17 animals per group in total. For dot plots, each dot represents one animal. *, p<0.05, **, p<0.01, ***, p<0.001, ****, p<0.0001, ns = not significant. All data are shown as the median and interquartile range.

In order to determine if these cells remain functional despite PD-1 expression or perhaps even require PD-1 signaling to control EBV infection, we examined the effect of treating 10^5^ (high dose) EBV-infected huNSG mice with an anti-PD-1 antibody in order to block the PD-1 axis. We initiated anti-PD-1 treatment three weeks after infection, after initial T cell priming events were presumed to have taken place and which also represents the timepoint of clinical IM presentation, and administered this antibody at regular intervals until the end of the experiment at five weeks post-infection (Fig 7A). We observed that anti-PD-1 treated animals had significantly higher blood (Fig 7B) and spleen viral loads (Fig 7C), compared with control animals. Interestingly, anti-PD-1 treated animals also had significantly elevated tumor burdens, as illustrated by the frequency of animals with tumors (Fig 7D left) and by the number of tumors per animal (right). Animals treated with anti-PD-1 appeared to have increased splenomegaly relative to control animals (Fig 7E). The antibody administered during treatment appeared to block or lead to the internalization of the receptor, as staining with the same clone resulted in lower splenic (Fig 7F) and blood (Fig 7G) frequencies of PD-1, while total T cell numbers remained similar in blood (Fig 7H) and spleen (Fig 7I). Anti-PD-1 treatment also did not appear to influence central and effector memory CD8^+^ T cell expansion and, in particular, did not drive more cells into the terminal EMRA differentiation stage (Fig 7J). Therefore, altered T cell differentiation does not seem to account for the loss of immune control. We detected elevated cytokine levels upon PD-1 blockade (Fig 7K), including the immune suppressive cytokine IL-10, which could inhibit protective cytotoxicity by T cells during EBV infection to some extent. Therefore, the intact function of the PD-1 axis is necessary for more effective control of EBV infection and tumorigenesis.

### Protective expansion of PD-1^+^, Tim3^+^, and KLRG1^+^ CD8^+^ T cells peaks when viral loads decrease during long term low dose EBV infection

**Fig 8:**
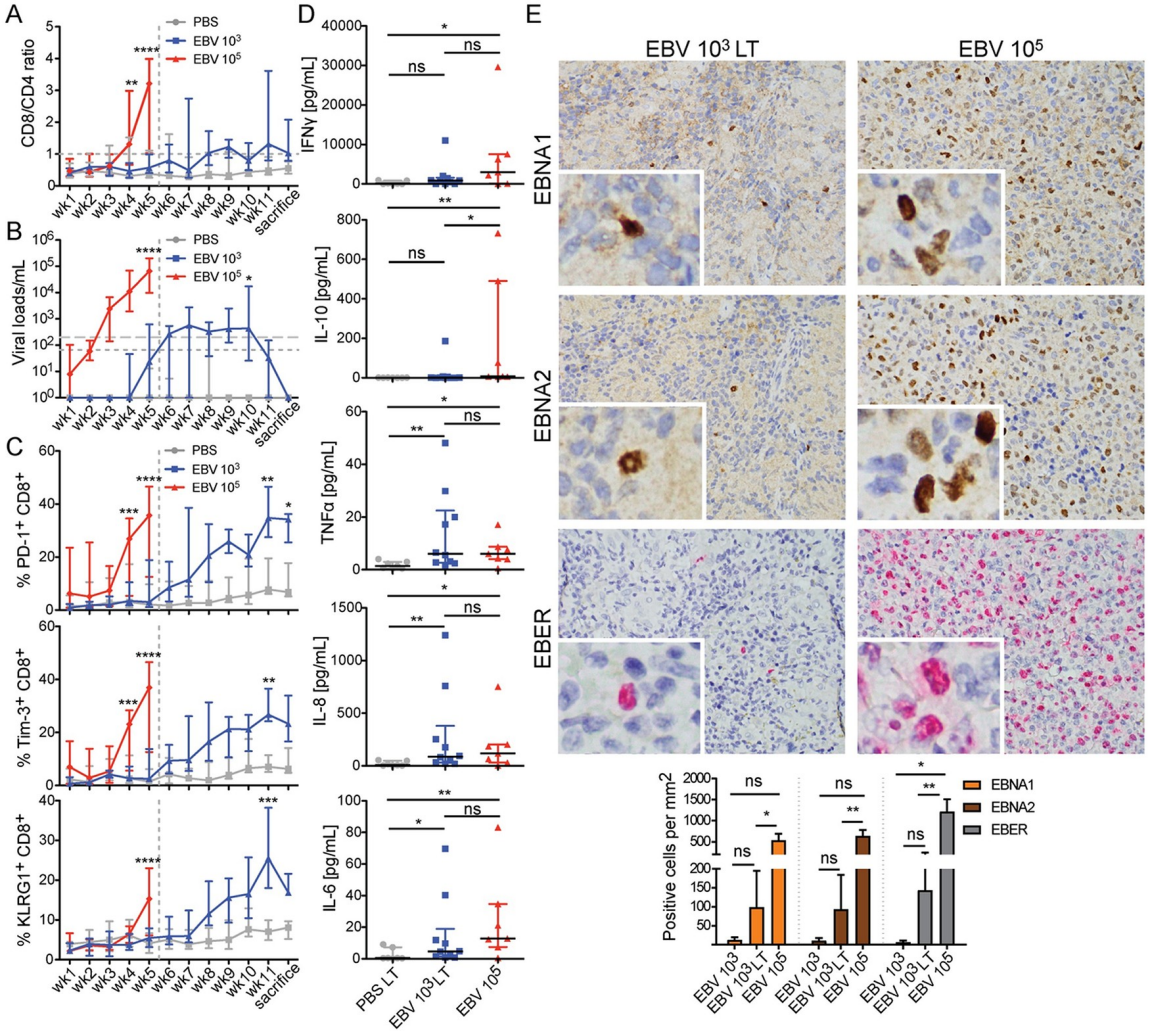
Protective expansion of PD-1^+^, Tim3^+^ and KLRG1^+^ CD8^+^ T cells peaks when viral loads decrease during long term low dose EBV infection. **A)** The ratio of CD8^+^ T cells relative to CD4^+^ T cells as a measure for CD8^+^ T cell expansion (n = 6–12 animals/group). **B)** Blood viral loads over time (n = 6–12 animals/group). Horizontal dotted line represents the lower limit of quantification (LLOQ) of EBV DNA copies and the dashed line represents 3 times the LLOQ. Mice with undetectable EBV DNA copies were plotted on the x-axis. **C)** Longitudinal data examining the expansion of PD-1^+^, Tim-3^+^, and KLRG1^+^ cells over time in blood, during an extended infection course with lower EBV dose. For A-C, data was analyzed with two-way ANOVA and Tukey’s correction was used as a post hoc test for week 1–5; Sidak’s correction was used for week 6 to sacrifice (the endpoint of the experiment). P values were compared between the EBV 10^3^ and EBV 10^5^ groups for week 1–5; the EBV 10^3^ and PBS group were compared from week 6 to sacrifice. **D)** Serum cytokines at the time of sacrifice. LT = long term. EBV 10^5^ animals were sacrificed at 5 weeks post-infection. Data were analyzed using the Kruskal-Wallis test (IFNγ: p = 0.0731, IL-10: p = 0.0037, TNFα p = 0.0147, IL-8 p = 0.0152, IL-6 p = 0.0098) and statistics from the Dunn’s post-test are displayed. **E)** 400x immunohistochemistry (EBNA1 and EBNA2) or in situ hybridization (EBER) images taken of representative EBV 10^3^ long term or EBV 10^5^ huNSG animals. Insets represent a further 4-fold zoom to display nuclear staining patterns. The graph on the bottom displays quantification of the number of positive cells per mm^2^ for the respective EBV and EBV latency markers during various infectious conditions. Kruskal-Wallis test with Dunn’s post-test was used for statistical analysis of the grouped EBNA1, EBNA2 and EBER conditions. Data are shown as the median and interquartile range. All data displayed in this figure were combined from 2 independent experiments (PBS: n = 6, EBV 10^3^ n = 12, EBV 10^5^ n = 7). All figures were displayed with the median and interquartile range. *, p<0.05, **, p<0.01, ***, p<0.001, and.****, p<0.0001.

To better understand the role of PD-1^+^ CD8^+^ T cells during in vivo protection, we observed their kinetics longitudinally during low dose EBV infection in huNSG mice. While CD8^+^ T cells rapidly expanded in the first five weeks with kinetics similar to IM [50] during high dose EBV infection, low dose EBV infection (10^3^ infectious particles) led to a gradual and more moderate increase in CD8^+^ T cell expansion, which plateaued at six to eight weeks after infection and seemed to decline after eleven weeks (Fig 8A). Viral titers also never reached 10^5^ genome equivalents/mL in the peripheral blood as during high dose EBV infection, but remained above the detection limit, with a peak of 10^4^ genome equivalents at ten weeks followed by a decline (Fig 8B). Interestingly, the frequency of PD-1, Tim-3, and KLRG1 positive CD8^+^ T cells gradually increases and reaches a similar level as during high dose infection after eleven weeks of infection (Fig 8C). This coincided with the decline in viral titers during low dose EBV infection. At three months post-infection with low dose EBV, serum levels were biased towards proinflammatory cytokines and chemokines like TNFα, IL-8, and IL-6, while IFNγ and IL-10 were not significantly elevated, in contrast to what we observed after five weeks of high dose EBV infection (Fig 8D). This improved immune control after twelve weeks of low dose EBV infection compared to five weeks of high dose EBV infection was also observed by immunohistochemistry in spleen sections of EBV infected huNSG mice (Fig 8E). As with the ten-fold difference in the peripheral blood viral loads, a ten-fold lower frequency of EBER^+^, EBNA1^+^, or EBNA2^+^ cells was attained after three months of low dose EBV infection compared to five weeks of high dose EBV infection (Fig 8E left vs. right column of micrographs and bar graph below). At five weeks of low dose EBV infection, EBV-infected cells were hardly detectable in the spleen of infected huNSG mice. This data indicates that IL-10 and IFNγ may not be particularly useful for viral clearance and that PD-1^+^, Tim3^+^, and KLRG1^+^ CD8^+^ T cells are important for controlling EBV infection.

## Discussion

In contrast to previous studies devoted to other virus systems such as murine LCMV, we find that the frequency of cells that express PD-1 and other inhibitory receptors appears to increase and is sustained upon EBV infection. These PD-1^+^ cells retain their proliferative capacity as gauged by Ki67, are able to produce important cytokines such as IFNγ and TNFα, and also retain their cytotoxic abilities. Co-expression of Tim3 and KLRG1 does not seem to significantly compromise these functions. Furthermore, CXCR5 and TCF1 expression, as well as the involvement of BATF3 by a subset of these triple positive CD8^+^ T cells might maintain their protective abilities during primary EBV infection.

Importantly, it appears that PD-1 signaling contributes to the immune control of EBV infection. Antibody blockade of this pathway starting at week 3 post-infection resulted in elevated viral loads, increased tumor burden, and elevated immunosuppressive cytokine production during primary EBV infection in huNSG mice. Our data strongly suggest that PD-1 blockade might not be advisable during primary EBV infection and that PD-1 positive CD8^+^ T cells contain subpopulations protective against persistent viral infections. Similarly, reactivation of two other pathogens was recently observed under PD-1 blockade treatment. Adult T-cell leukemia-lymphoma, associated with the human T cell lymphotropic virus (HTLV-1), progressed rapidly with increased viral replication under anti-PD-1 therapy [53]. Furthermore, mycobacterium tuberculosis dissemination occurred in patients with PD-1 blockade, possibly due to increased immunopathology by elevated Mtb specific CD4^+^ T cells responses [54]. Thus, several important human pathogens appear to reactivate than become better controlled upon inhibition of the PD-1 pathway.

In contrast to our study, Ma et. al observed that upon early intervention with both PD-1 and CTLA4 blocking antibodies, tumor burdens in their EBV-infected cord blood reconstituted huNSG model were diminished [55]. The humanized mouse model that was used in that study does not promote efficient EBV specific immune control and 80% of the EBV infected mice develop B cell lymphomas [56]. The B cell compartment in that model preferentially supports tumor formation in a T cell dependent manner, as can be observed during LMP1-deficient EBV infection. Infection with LMP1 deficient virus causes similar levels of lymphomagenesis as wild-type virus infection, but tumor formation is abolished after CD4^+^ T cell depletion [56]. In contrast, the mouse model reported here (using uninfected hematopoietic progenitor cells to reconstitute huNSG mice) develops protective T cell responses after EBV infection [18, 19, 57]. Using this model, we demonstrate in the current study that PD-1 positive CD8^+^ T cells appear to contribute to this protection.

A recent study by the Wherry laboratory compared PD-1 positive CD8^+^ T cell subsets in several human viral infections, including HIV, influenza A virus, and human cytomegalovirus (HCMV) [35]. Interestingly, these different infections promoted quite different PD-1 positive CD8^+^ T cell populations. Acute influenza A virus infection seemed to establish mainly central memory CD8^+^ T cells and a PD-1^+^2B4^+^CXCR5^+^ compartment after clearance. In contrast, viremic HIV infected individuals seemed to lack this compartment entirely. Our data suggest that acute EBV infection, despite persistence of the virus, allows for a significant central memory expansion both in IM and high dose EBV infected huNSG mice. Moreover, we detected PD-1^+^2B4^+^CXCR5^+^ CD8^+^ T cells that expand during primary EBV infection. Therefore, the human immune system manages, despite high EBV loads during IM and high dose EBV infection in huNSG mice, to establish protective PD-1 positive T cell compartments, which are lost in ill-controlled viral infections such as HIV.

The precise role that PD-1 plays in these T cell populations remains unclear. However, in the case of symptomatic primary EBV infection, we observe elevated levels of multiple serum cytokines and chemokines that might be mainly responsible for the immune pathology during IM [44]. We propose that PD-1 signaling might curb this deleterious cytokine storm in order for cytotoxic T cell functions to efficiently control EBV infection and associated tumorigenesis. It will be interesting to understand how the respective protective PD-1 positive CD8^+^ T cell populations could be induced in patients that suffer from EBV associated malignancies. The results from this study indicate that monitoring of PD-1 positive CD8^+^ T cell induction during future EBV specific vaccination and immunotherapy studies may inform patient outcomes.

## Materials and methods

### Selection of patients and healthy controls

Acutely infected patients at the University Children’s Hospital of Zurich were included in the study based on clinical features suggestive of IM (tonsillitis/angina, adenopathy/swollen lymph nodes, and fever) and the exclusion of Streptococcus A by rapid swab. The diagnosis was confirmed by serology performed on blood drawn upon admittance to the hospital (EBV VCA IgM^+^, VCA IgG^+^, EBNA IgG^-^). EBV DNA loads in plasma were quantified using qPCR. Healthy EBV seropositive children who underwent elective tonsillectomy at the University Children’s Hospital of Zurich and healthy EBV seropositive adults were used as controls. All patients and healthy donors were negative for CMV IgM, indicating that they were not acutely infected with CMV (S1E Fig). Written informed consent was obtained from patients and healthy donors, and samples were anonymized.

### Recombinant Epstein-Barr virus (EBV)

EBV strain B95-8 was produced from human embryonic kidney HEK293 cells containing wild-type BACs (kind gift from H. Delecluse). This GFP expressing virus was titrated on Raji cells (ATCC, Manassus, VA, USA), and the frequency of GFP-expressing cells was analyzed by flow cytometry (BD FACSCantoII) two days post-infection to calculate Raji-infectious units (RIU).

### Humanized mouse generation and infection

NOD-scid γ_c_^-/-^ animals with or without the HLA-A2 transgene (Jackson Laboratory, Bar Harbor, Maine, USA) were maintained under specific pathogen-free conditions. Newborn mice up to five days of age were sublethally irradiated (1 Gy) and intrahepatically reconstituted with approximately 1–3 x 10^5^ HLA-A2^+^ CD34^+^ hematopoietic progenitor cells purified from human fetal liver tissue (Advanced Bioscience Resources). After twelve weeks, peripheral blood reconstitution of human CD45^+^ CD19^+^ B, CD3^+^ T, and NKp46^+^ NK cells, as well as CD4^+^ and CD8^+^ T cells were examined by flow cytometry. After engraftment and immunophenotyping, animals were intraperitoneally infected with either 10^3^ or 10^5^ EBV RIU, and followed for up to twelve weeks post-infection.

### Viral load quantification

Splenic tissue was processed and DNA isolated using the DNeasy Blood and Tissue Kit (QIAGEN), and total DNA from whole blood or plasma was extracted using the NucliSENS EasyMAG System (bioMérieux), according to manufacturer’s recommendations. TaqMan real-time PCR (Applied Biosystems) was used to quantify EBV DNA using modified primers for the BamH1 W fragment (5′-CTTCTCAGTCCAGCGCGTTT-3′ and 5′-CAGTGGTCCCCCTCCCTAGA-3′) and a fluorogenic probe (5′-FAM CGTAAGCCAGACAGCAGCCAATTGTCAG-TAMRA-3′). Samples were analyzed in duplicates.

### Flow cytometry, antibody and pentamer labeling

For surface stainings, antibodies were incubated together with cells for 20 minutes at 4°C, followed by a wash and fixation in 1% paraformaldehyde (PFA). For intracellular labelings, surface labelings were performed for 20 minutes at 4°C, followed by fixation in 1% PFA for 20 minutes. This was followed by at least two washes in PBS+0.05% saponin (PS). Intracellular antibodies were diluted in PS and cells labeled for 20 minutes at 4°C. Cells were washed once in PBS. Intranuclear stainings were done following the instructions given in the FoxP3/Transcription Factor Staining Buffer Set (eBioscience, 00-5523-00). All samples were acquired using the DIVA software on either a BD FACSCantoII or a BD LSRFortessa, and analysis was performed using FlowJo software (Treestar).

Antibody clones used in this study: 2B4 (C1.7, Biolegend), BTLA (J168-540, BD Biosciences), CD3 (OKT3, Biolegend), CD4 (OKT4, Biolegend), CD8 (SK1, Biolegend), CD19 (HIB19, Biolegend), CD27 (M-T271, BD Biosciences), CD28 (CD28.2, BD Biosciences), CD45 (HI30, Biolegend), CD45RA (HI100, Biolegend), CD62L (DREG-56, BD Biosciences), CD107a (H4A3, BD Biosciences), CD127 (A019D5, Biolegend), CTLA4 (BNI3, BD Biosciences), Granzyme B (GB11, Biolegend), HLA-DR (G46-6, L243, Biolegend), IFNγ (4S.B3, eBioscience), IL-2 (MQ1-17H12, eBioscience), Ki67 (20Raj1, eBioscience), KLRG1 (13F12F2, eBioscience), Lag-3 (17B4, Enzo), muTCR β chain (H57-597, Biolegend), NKp46 (9E2, BD Biosciences), PD-1 (EH12.2H7, NAT105, Biolegend), PD-L1 (29E.2A3, Biolegend), Tim-3 (344823, R&D), TNFα (Mab11, eBioscience).

For PD-1 blocking experiments, we intraperitoneally injected the PD-1 EH12.2H7 clone (Biolegend) or the control at the indicated amounts, according to the timeline in Fig 7.

HLA-A2^+^ EBV-pentamers specific for BMLF1 and LMP2 were purchased from Proimmune. Pentamers were added ten minutes in advance of surface antibody labeling and cells were processed as for normal surface stainings.

### Cell isolation

PBMCs were obtained from humanized mice by lysing blood for five minutes with 1x ACK lysis buffer, followed by washing. Spleens were manually dissociated, and cells filtered through a 70 μm strainer before separation of white blood cells on Ficoll-Paque gradients. Cells were counted using a Beckman Coulter AcT diff Analyzer. CD8^+^ and CD19^+^ cells were isolated via positive selection using Miltenyi microbeads and following manufacturer’s recommendations.

### Retroviral production, TCR transduction, and cell transfer

PhAmpho packaging cells were transfected using Fugene HD (Roche) with the pCL-Ampho construct and either LMP2-TCR or BMLF1-TCR construct, according to manufacturers’ instructions. After 24 hours, the viral supernatant was harvested from the transfected PhAmpho cells and frozen at -80°C.

Animals were euthanized and splenocytes were isolated as outlined above. Cells were stimulated with CD3/CD28 Dynabeads (Thermofisher Scientific) for 72 hours. During this time, non-TC treated 24-well plates (Greiner) were coated with retronectin (Takara) overnight at 4°C the day before spinfection. Retronectin was removed from plates, which were then washed once with PBS and blocked with 2% BSA in PBS for 30 minutes at room temperature. Dynabeads were removed from activated splenocytes, and cell were counted and plated into retronectin coated plates at 1 million cells/well. Retroviral supernatants containing either BMLF1-TCR or LMP2-TCR packaged retroviruses were thawed and activated splenocytes were centrifuged for one hour at 800 x g in the presence of these viruses. Cells were incubated together with IL-2 (20 U/mL, Peprotech), IL-7 (1 ng/mL, Miltenyi), and IL-15 (10 ng/mL, Peprotech), and the medium was changed the following day. Transduction efficiency was assessed by flow cytometry at the end of day 2. The following day, 200’000 TCR^+^ CD3^+^ T cells were transferred intravenously into donor-mate animals (animals reconstituted with the same CD34^+^ donor). Animals were assessed longitudinally during the course of the experiments for TCR^+^ CD3^+^ T cells.

### Plasma/Serum cytokine detection

Plasma or serum samples were thawed on ice and centrifuged to remove particulates. Samples were assayed for proinflammatory cytokines, chemokines, and other factors using the Meso Scale Diagnostics (Rockville, MD) V-plex (multiplex) platform and following manufacturer’s instructions.

### Ex vivo and transduced cell cytokine stimulation assay

Ex vivo isolated and rested CD8^+^ T cells or transduced T cells from splenocytes were incubated with (R10) alone (RPMI 1640 + 10% FBS + 1% penicillin/streptomycin + L-glutamine; Gibco, Thermo Fisher Scientific), EBV-specific peptides (BMLF1: GLCTLVAML; LMP2: CLGGLLTMV), or R10 containing PMA/ionomycin (17 ng/ml and 4 μM, respectively) for 2 hours, followed by the addition of brefeldin A and further incubation for an additional 6 hours. Cells were stained for intracellular cytokines and acquired on a BD LSRFortessa. For CD107a labeling, this antibody was added at the very start of coculture.

### Immunohistochemistry and in-situ hybridization

Splenic sections were excised, fixed in 4% formalin for 2–3 days, and paraffin embedded (SophistoLab). Three micron sections were cut and immunohistochemistry was performed with monoclonal antibodies directed against EBNA1 (clone 1H4, kind gift from Dr. Kremmer, Munich, Germany) and EBNA2 (clone PE2, Abcam) on a Leica BOND-III automated immunohistochemistry system. Diaminobenzidin (DAB) (Zytomed Systems, Berlin, Germany) was used as a chromogen. In-situ-hybridisation for detection of Epstein–Barr virus-encoded small RNAs (EBER) was performed as described previously [58] using Permanent Red/AP as a chromogen. Numbers of labelled cells were counted using an Olympus BX46 microscope with a DP27 camera. Except for cases with abundant positive cells, the whole tissue area was evaluated (at least 1 mm^2^).

### RNA isolation and microarray analysis

CD8^+^ T cells were isolated from Ficoll (GE Healthcare) separated PBMCs (human patient samples) or splenocytes (huNSG mice) using positive selection CD8^+^ microbeads from Miltenyi, following the manufacturer’s protocol. 0.5-1x10^6^ cells were snap frozen in Trizol (Thermo Fisher Scientific), and RNA was extracted using the Absolutely RNA microprep kit. cRNA was prepared and hybridized to an Agilent SurePrint G3 Human Gene Expression 8x60K v2 Microarray kit (Agilent Technologies) following the manufacturer’s protocol. The raw expression array signals were log-transformed and normalized using quantile normalization and probes that could not be matched to Entrez Gene identifiers were removed. limma (linear models for microarray analysis) was used to fit a linear model for each gene; linear contrasts for differences of interest were tested using moderated t-statistics [59] and corresponding p-values were adjusted using the Benjamini-Hochberg correction [60]. Gene set testing was done using the CAMERA package [61], using a subset of the MSigDB gene sets collection [62]. Gene lists in Fig 3 were generated by comparing genes that were up or downregulated in both IM patient and huNSG mice infected with 10^5^ infectious EBV comparisons. Full microarray data can be found in S1 Table.

### tSNE and FlowSOM analysis

Data were acquired with a BD LSR-Fortessa flow cytometer, compensated, exported into FlowJo software (version 9, TreeStar Inc.). The exported FCS files were normalized using Cyt MATLAB (version 2017b) and uploaded into Rstudio (R software environment, version 3.4.0). tSNE and FlowSOM algorithm mapping live T cells from a pooled sample were performed as described by Nowicka et al. [63].

### Statistical analysis

Statistical analysis was performed using GraphPad Prism software. The two-tailed Mann-Whitney U test was used to analyze pair-wise data, and correlations on non-parametric data were assessed using the Spearman’s rank correlation coefficient. The one-way ANOVA (Kruskal-Wallis test) followed by Dunn’s post test was used to compare non-parametric data sets with 3 or more conditions. The two-way ANOVA test was used to compare the differences between different conditions with two independent variables. Sidak’s post hoc analysis was used when two conditions were compared; alternatively, Tukey’s post hoc analysis was used when three conditions were compared. A p value < 0.05 was considered statistically significant.

### Ethics statement and animal protocols

The cantonal ethical committee of Zurich, Switzerland (protocol KEK-StV-Nr.19/08) approved studies involving human fetal liver samples as well as patient and health volunteer PBMCs. Research was conducted in accordance with the Declaration of Helsinki. All described animal experimentation was approved by the cantonal veterinary office of Zurich, Switzerland (protocols 148/2011, ZH209_2014 and ZH159_17) and conducted according to the Swiss Animal Welfare Act, Tierschutzgesetz (TSchG).

## Supporting information

S1 Fig Characteristics of IM patients and their T cells. **A)** Donor ages, displayed together with the median. **B-D)** Viral load and indicated receptor correlations in IM patients. Each point represents one donor. Data were analyzed using the nonparametric Spearman correlation. E) Table indicating the serological test results of IM patients and healthy donors.
(PDF)

S2 Fig Reconstitution of human immune system components in NSG mice. **A)** Scheme depicting the reconstitution workflow and data from 4 independent experiments used showing the relative reconstitution frequencies of T cells (CD3, CD4, CD8) as well as of B cells and NK cells. Each point represents one reconstituted mouse. **B)** Timeline of EBV infection in huNSG animals.
(PDF)

S3 Fig Expression of inhibitory and differentiation molecules of huCD45^+^ cells. **A)** tSNE analysis of huCD45^+^ cells from huNSG animals examining PD-1, CD244 (2B4), BTLA, and CD127 expression in the context of different cell types (monocytes, CD8^+^ T, CD4^+^ T and CD19^+^ B cells as indicated by arrows). **B)** As in A), tSNE analysis of huCD45^+^ cells from huNSG animals but examining PD-1, KLRG1, Tim-3, and CD127 expression in the context of different immune cell types.
(PDF)

S4 Fig Transduced splenocytes respond to their cognate peptides. **A)** Scheme for generation and transfer of EBV-specific T cells, followed by infection. **B)** Peptide-specific responses for BMLF1 TCR transduced cells (top) and LMP2 TCR transduced cells (bottom). The irrelevant peptide is either the A2-restricted LMP2 peptide for BMLF1 transduced cells, or the A2-restricted BMLF1 peptide for LMP2 transduced cells. One representative experiment of 2–3 experiments. Data are displayed as median and interquartile range.
(PDF)

S5 Fig IM patients and huNSG mice infected with EBV retain unique transcriptional characteristics. **A)** Microarray data from Fig 3 examining genes found in the GO term for T cell mediated cytotoxicity (GO:0001913). Data are separated by species. **B)** Microarray data from Fig 3 examining genes found in the GO term for T cell costimulation (GO:0031295), separated by species.
(PDF)

S6 Fig Cytokines, chemokines, and other factors are found in IM patient plasma and huNSG mouse serum. **A)** Plasma cytokines from IM patients. Each dot represents one donor. Data were analyzed using the Mann-Whitney U test. **B-D)** Proinflammatory cytokines, chemokines, and other factors found in the serum of PBS treated or EBV infected huNSG animals at the time of sacrifice. Data were analyzed using the Kruskal-Wallis test, and the results of the Dunn’s post-test are displayed. Each point represents one animal, and data are displayed using the median and interquartile range. Data were combined from 2–4 independent experiments. *, p<0.05, **, p<0.01, and ns = not significant.
(PDF)

S7 Fig PD-1^+^ CD8^+^ T cells co-express multiple inhibitory and differentiation receptors and retain functionality. **A)** tSNE analysis of PD-1, CD244 (2B4), BTLA, CD127, CXCR5, and CD45RA co-expression within the CD8^+^ population, where red indicates higher expression. **B)** Cell clustering analysis of the data from A), comparing PBS and high dose EBV conditions in huNSG animals and the frequencies of inhibitory and differentiation receptor containing populations in a tSNE plot (top), and graphically (bottom). **C)** tSNE analysis of the CD8^+^ T cell population examining the coexpression of PD-1 and CD45RA together with CD107a, Granzyme B, and IFNγ.
(PDF)

S8 Fig Treatment with anti-PD-1 antibodies results in higher levels of proinflammatory cytokines. **A-C)** Serum cytokines at the time of sacrifice. Data were analyzed using the Kruskal-Wallis test (IL-6: p = 0.0004, IL-2: p = 0.5890, IL-1β: p = 0.0317, IL-4: p = 0.0106), and statistics from the Dunn’s post-test are displayed. In all panels, data displayed were combined from 3 independent experiments, with 5–17 animals per group in total. Each point represents one animal. Data are shown as the median and interquartile range. *, p<0.05, **, p<0.01, ns = not significant.
(PDF)

S1 Table Gene expression of IM patients and huNSG mice infected with EBV. (XLSX)
