# Supplementary material for: CD8+ T cells retain protective functions despite sustained inhibitory receptor expression during Epstein-Barr virus infection in vivo
Source: PLoS Pathog. 2019 May 30;15(5):e1007748. doi: 10.1371/journal.ppat.1007748 (PMC6542544; doi:10.1371/journal.ppat.1007748)
Supplement: S1 Fig — A) Donor ages, displayed together with the median. B-D) Viral load and indicated receptor correlations in IM patients. Each point represents one donor. Data were analyzed using the nonparametric Spearman correlation. E) Table indicating the serological test results of IM patients and healthy donors. (PDF) [file ppat.1007748.s001.pdf]

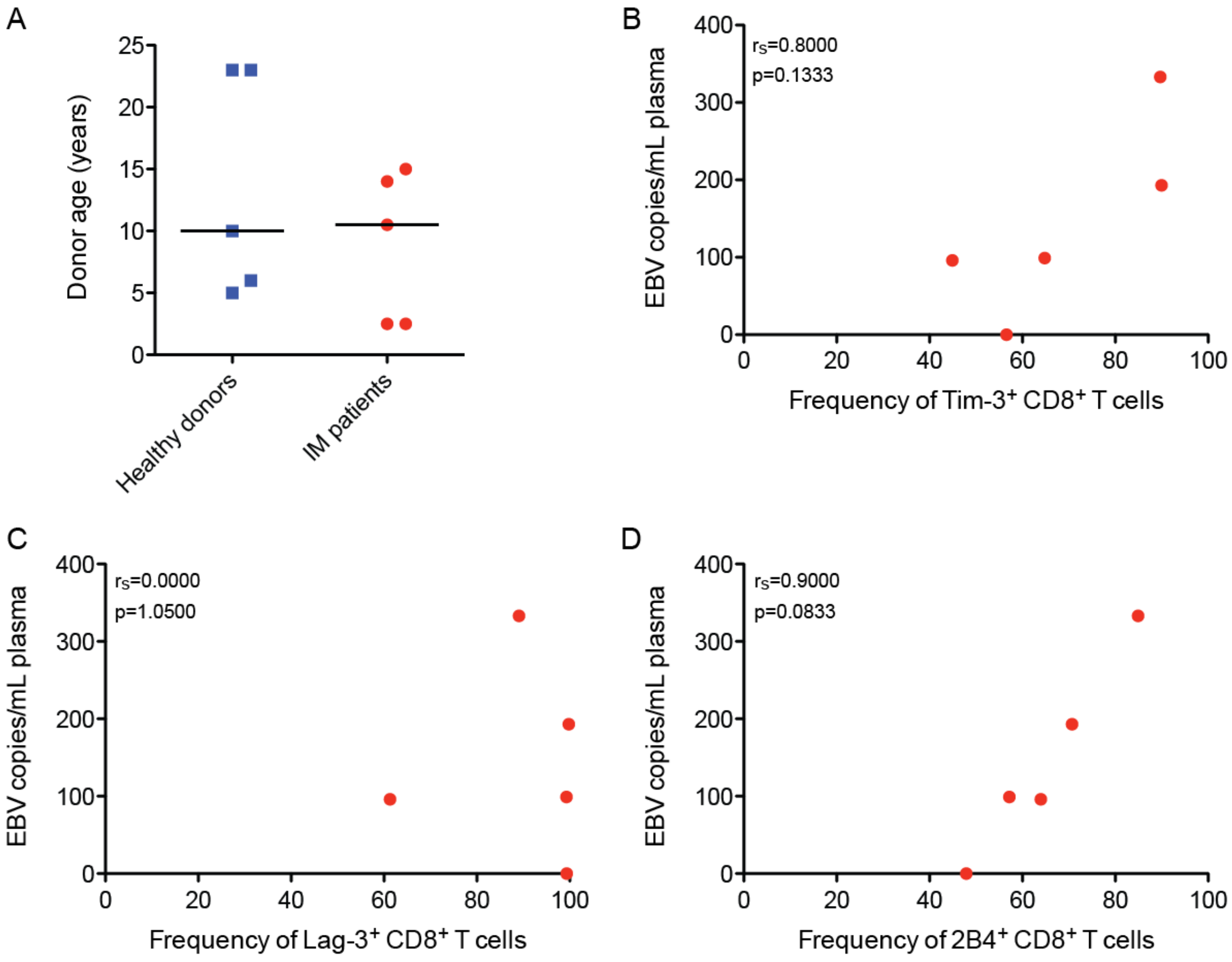

E

		IM1	IM2	IM3	IM4	IM5		HD1	HD2	HD3	HD4	HD5
**IgG**	EBV-VCA	+	+	+	+	+		+	+	+	+	+
	EBNA-1	-	-	-	-	-		-	-	+	-	-
	CMV	+	-	-	+	-		-	-	+	-	-
	T. gondii	-	-	-	-	-		-	-	-	-	-
**IgM**	Heterophile-1	+	+	+	-	+		-	-	-	-	-
	Heterophile-2	+	+	+	-	+		-	-	-	-	-
	EBV-VCA	+	+	+	+	+		-	-	-	-	-
	CMV	-	-	-	-	-		-	-	-	-	-

Figure S1: Characteristics of IM patients and their T cells. A) Donor ages, displayed together with the median. **B-D)** Viral load and indicated receptor correlations in IM patients. Each point represents one donor. Data were analyzed using the nonparametric Spearman correlation. **E)** Table indicating the serological test results of IM patients and healthy donors.
