# Supplementary material for: CD8+ T cells retain protective functions despite sustained inhibitory receptor expression during Epstein-Barr virus infection in vivo
Source: PLoS Pathog. 2019 May 30;15(5):e1007748. doi: 10.1371/journal.ppat.1007748 (PMC6542544; doi:10.1371/journal.ppat.1007748)
Supplement: S2 Fig — A) Scheme depicting the reconstitution workflow and data from 4 independent experiments used showing the relative reconstitution frequencies of T cells (CD3, CD4, CD8) as well as of B cells and NK cells. Each point represents one reconstituted mouse. B) Timeline of EBV infection in huNSG animals. (PDF) [file ppat.1007748.s002.pdf]

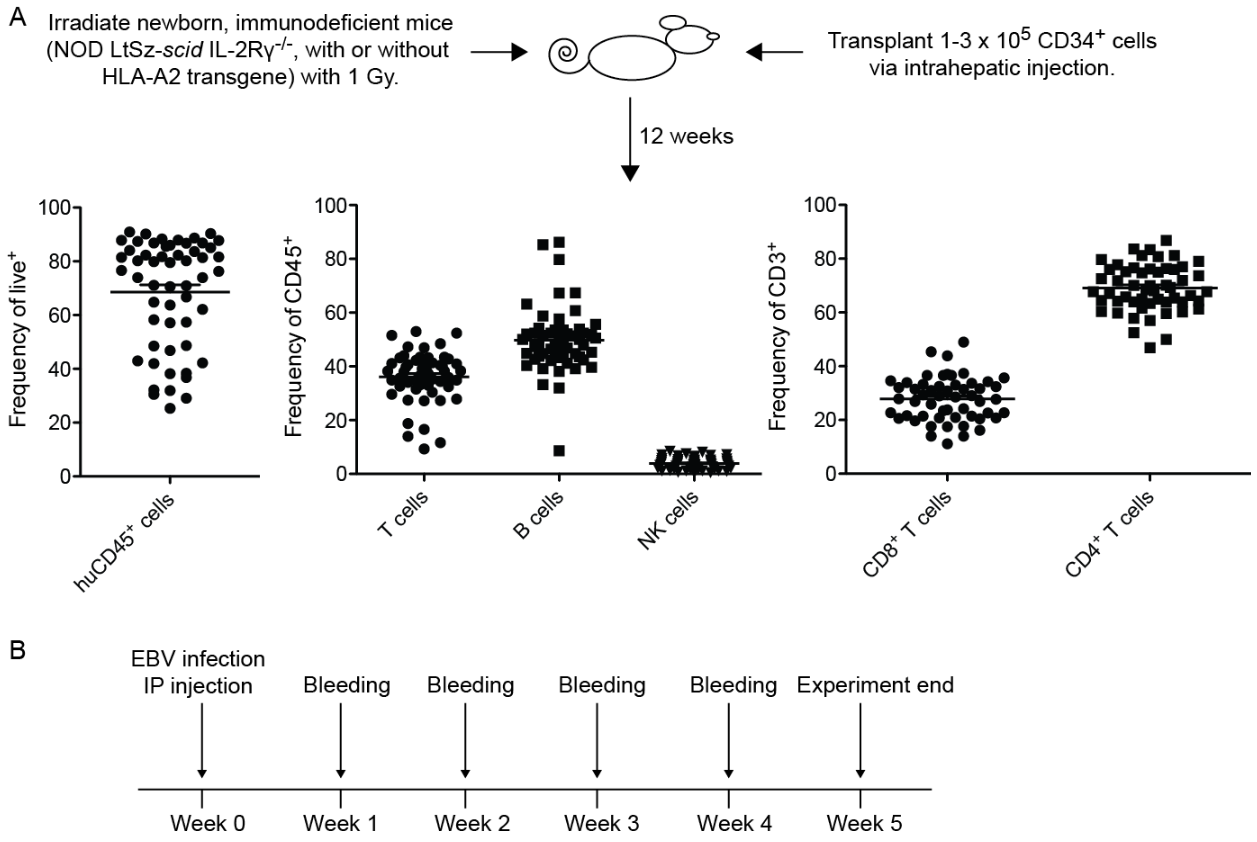

Figure S2: Reconstitution of human immune system components in NSG mice. A) Scheme depicting the reconstitution workflow and data from 4 independent experiments used showing the relative reconstitution frequencies of T cells (CD3, CD4, CD8) as well as of B cells and NK cells. Each point represents one reconstituted mouse. **B)** Timeline of EBV infection in huNSG animals.
