# Supplementary material for: CD8+ T cells retain protective functions despite sustained inhibitory receptor expression during Epstein-Barr virus infection in vivo
Source: PLoS Pathog. 2019 May 30;15(5):e1007748. doi: 10.1371/journal.ppat.1007748 (PMC6542544; doi:10.1371/journal.ppat.1007748)
Supplement: S3 Fig — A) tSNE analysis of huCD45+ cells from huNSG animals examining PD-1, CD244 (2B4), BTLA, and CD127 expression in the context of different cell types (monocytes, CD8+ T, CD4+ T and CD19+ B cells as indicated by arrows). B) As in A), tSNE analysis of huCD45+ cells from huNSG animals but examining PD-1, KLRG1, Tim-3, and CD127 expression in the context of different immune cell types. (PDF) [file ppat.1007748.s003.pdf]

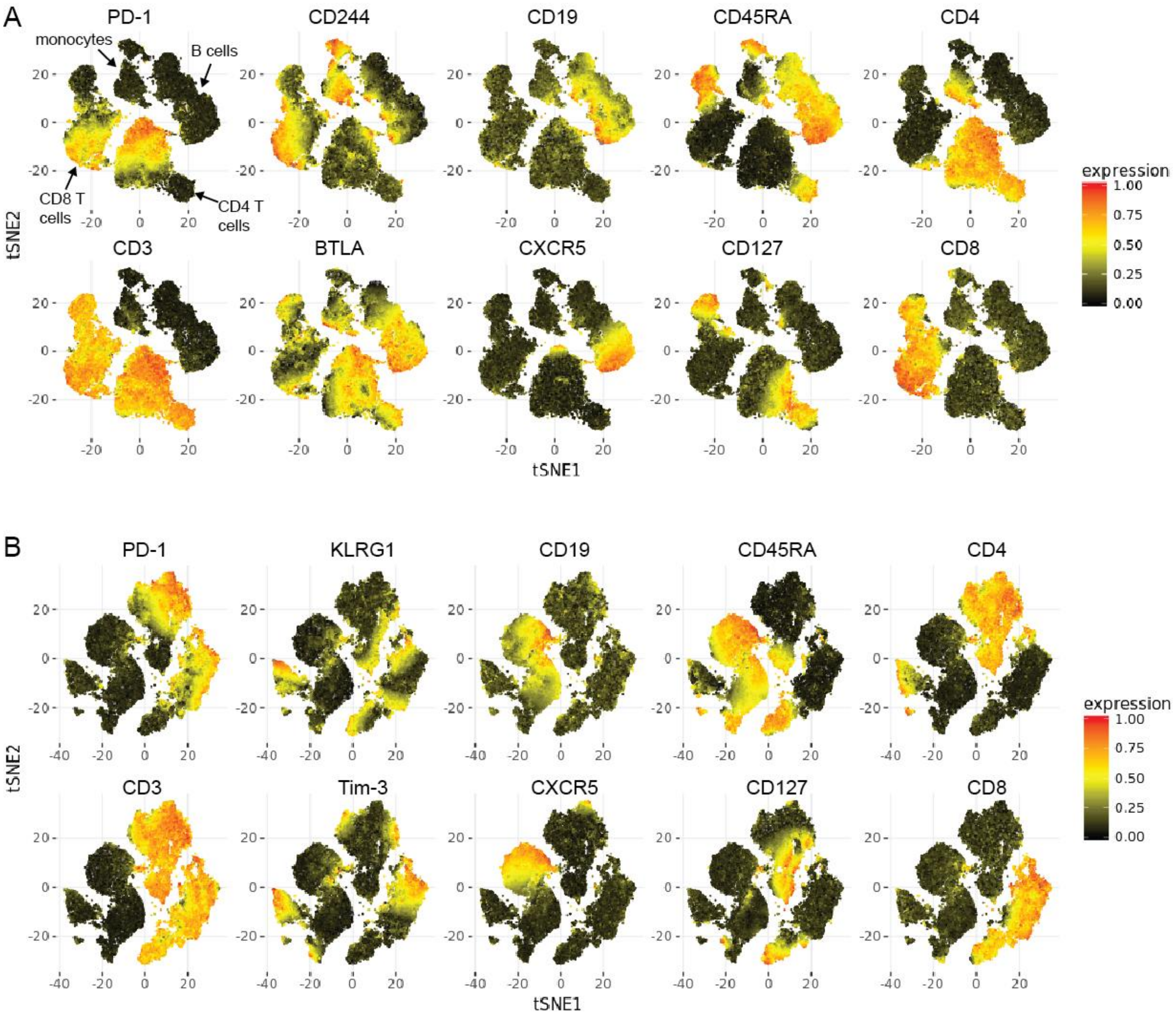

Figure S3: Expression of inhibitory and differentiation molecules of huCD45⁺ cells. A) tSNE analysis of huCD45⁺ cells from huNSG animals examining PD-1, CD244 (2B4), BTLA, and CD127 expression in the context of different cell types (monocytes, CD8⁺ T, CD4⁺ T and CD19⁺ B cells as indicated by arrows). **B)** As in A), tSNE analysis of huCD45⁺ cells from huNSG animals but examining PD-1, KLRG1, Tim-3, and CD127 expression in the context of different immune cell types.
