# Supplementary material for: CD8+ T cells retain protective functions despite sustained inhibitory receptor expression during Epstein-Barr virus infection in vivo
Source: PLoS Pathog. 2019 May 30;15(5):e1007748. doi: 10.1371/journal.ppat.1007748 (PMC6542544; doi:10.1371/journal.ppat.1007748)
Supplement: S4 Fig — A) Scheme for generation and transfer of EBV-specific T cells, followed by infection. B) Peptide-specific responses for BMLF1 TCR transduced cells (top) and LMP2 TCR transduced cells (bottom). The irrelevant peptide is either the A2-restricted LMP2 peptide for BMLF1 transduced cells, or the A2-restricted BMLF1 peptide for LMP2 transduced cells. One representative experiment of 2–3 experiments. Data are displayed as median and interquartile range. (PDF) [file ppat.1007748.s004.pdf]

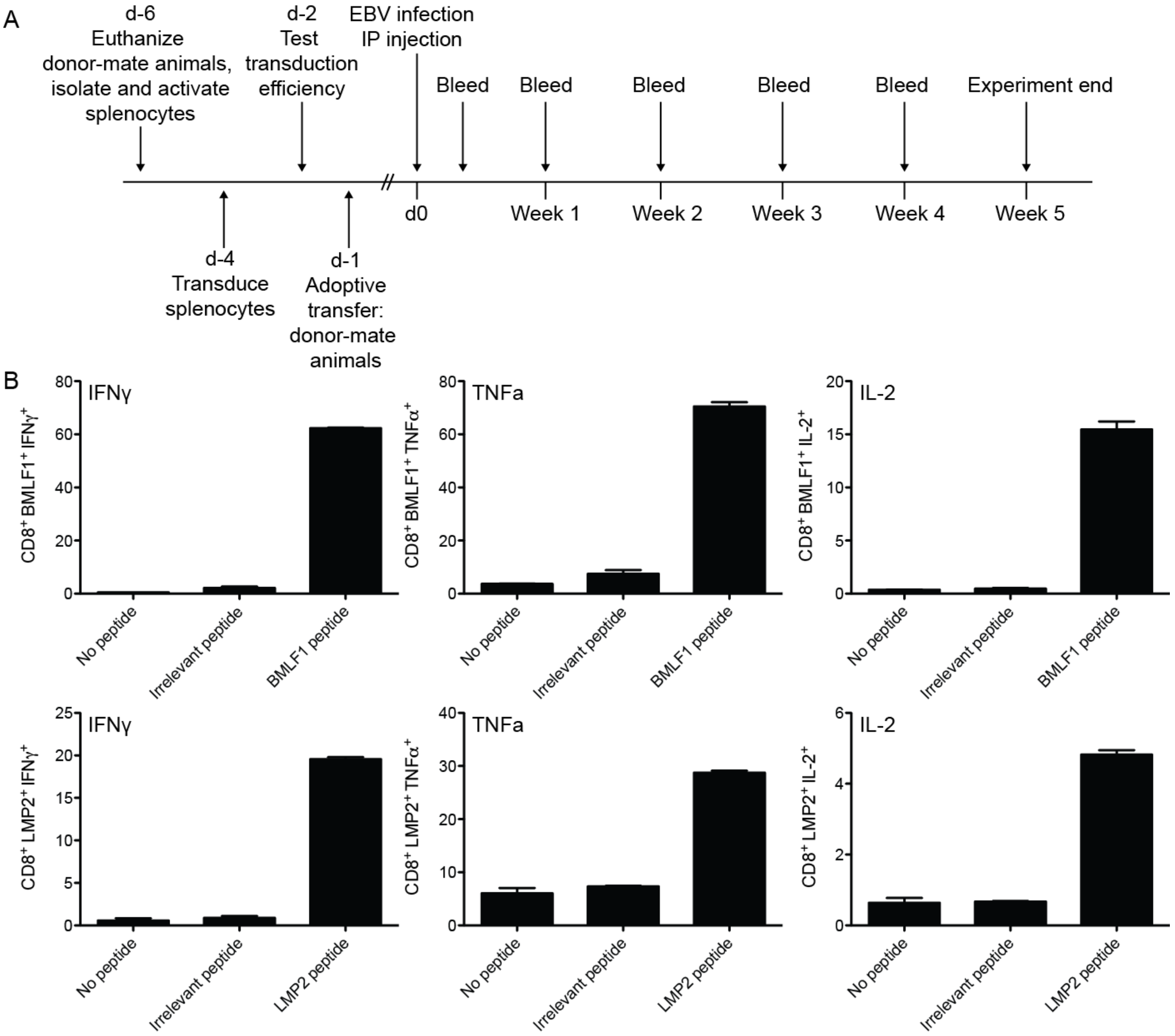

Figure S4: Transduced splenocytes respond to their cognate peptides. A) Scheme for generation and transfer of EBV-specific T cells, followed by infection. **B)** Peptide-specific responses for BMLF1 TCR transduced cells (top) and LMP2 TCR transduced cells (bottom). The irrelevant peptide is either the A2-restricted LMP2 peptide for BMLF1 transduced cells, or the A2-restricted BMLF1 peptide for LMP2 transduced cells. One representative experiment of 2-3 experiments. Data are displayed as median and interquartile range.
