# Supplementary figures and images for: CD8+ T cells retain protective functions despite sustained inhibitory receptor expression during Epstein-Barr virus infection in vivo
Source: PLoS Pathog. 2019 May 30;15(5):e1007748. doi: 10.1371/journal.ppat.1007748 (PMC6542544; doi:10.1371/journal.ppat.1007748)

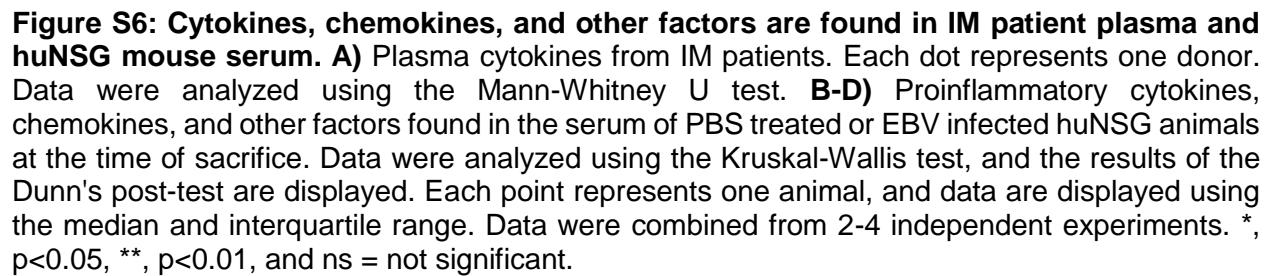

Supplement: S6 Fig — A) Plasma cytokines from IM patients. Each dot represents one donor. Data were analyzed using the Mann-Whitney U test. B-D) Proinflammatory cytokines, chemokines, and other factors found in the serum of PBS treated or EBV infected huNSG animals at the time of sacrifice. Data were analyzed using the Kruskal-Wallis test, and the results of the Dunn’s post-test are displayed. Each point represents one animal, and data are displayed using the median and interquartile range. Data were combined from 2–4 independent experiments. *, p<0.05, **, p<0.01, and ns = not significant. (PDF) [file ppat.1007748.s006.pdf]
