# Supplementary material for: CD8+ T cells retain protective functions despite sustained inhibitory receptor expression during Epstein-Barr virus infection in vivo
Source: PLoS Pathog. 2019 May 30;15(5):e1007748. doi: 10.1371/journal.ppat.1007748 (PMC6542544; doi:10.1371/journal.ppat.1007748)
Supplement: S7 Fig — A) tSNE analysis of PD-1, CD244 (2B4), BTLA, CD127, CXCR5, and CD45RA co-expression within the CD8+ population, where red indicates higher expression. B) Cell clustering analysis of the data from A), comparing PBS and high dose EBV conditions in huNSG animals and the frequencies of inhibitory and differentiation receptor containing populations in a tSNE plot (top), and graphically (bottom). C) tSNE analysis of the CD8+ T cell population examining the coexpression of PD-1 and CD45RA together with CD107a, Granzyme B, and IFNγ. (PDF) [file ppat.1007748.s007.pdf]

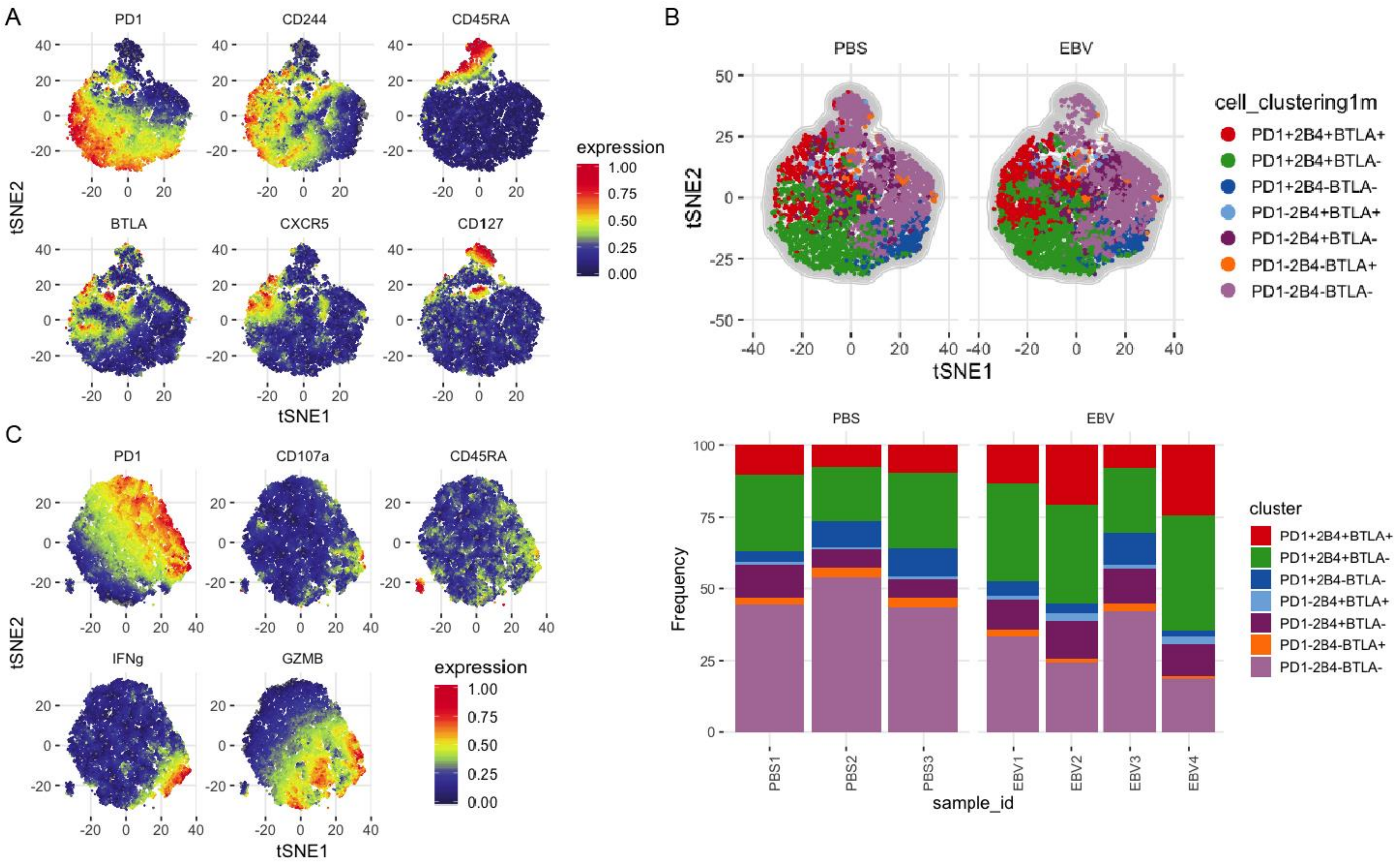

Figure S7: PD-1⁺ CD8⁺ T cells co-express multiple inhibitory and differentiation receptors and retain functionality. A) tSNE analysis of PD-1, CD244 (2B4), BTLA, CD127, CXCR5, and CD45RA co-expression within the CD8⁺ population, where red indicates higher expression. **B)** Cell clustering analysis of the data from A), comparing PBS and high dose EBV conditions in huNSG animals and the frequencies of inhibitory and differentiation receptor containing populations in a tSNE plot (top), and graphically (bottom). **C)** tSNE analysis of the CD8⁺ T cell population examining the coexpression of PD-1 and CD45RA together with CD107a, Granzyme B, and IFNγ.
