# Supplementary material for: CD8+ T cells retain protective functions despite sustained inhibitory receptor expression during Epstein-Barr virus infection in vivo
Source: PLoS Pathog. 2019 May 30;15(5):e1007748. doi: 10.1371/journal.ppat.1007748 (PMC6542544; doi:10.1371/journal.ppat.1007748)
Supplement: S8 Fig — A-C) Serum cytokines at the time of sacrifice. Data were analyzed using the Kruskal-Wallis test (IL-6: p = 0.0004, IL-2: p = 0.5890, IL-1β: p = 0.0317, IL-4: p = 0.0106), and statistics from the Dunn’s post-test are displayed. In all panels, data displayed were combined from 3 independent experiments, with 5–17 animals per group in total. Each point represents one animal. Data are shown as the median and interquartile range. *, p<0.05, **, p<0.01, ns = not significant. (PDF) [file ppat.1007748.s008.pdf]

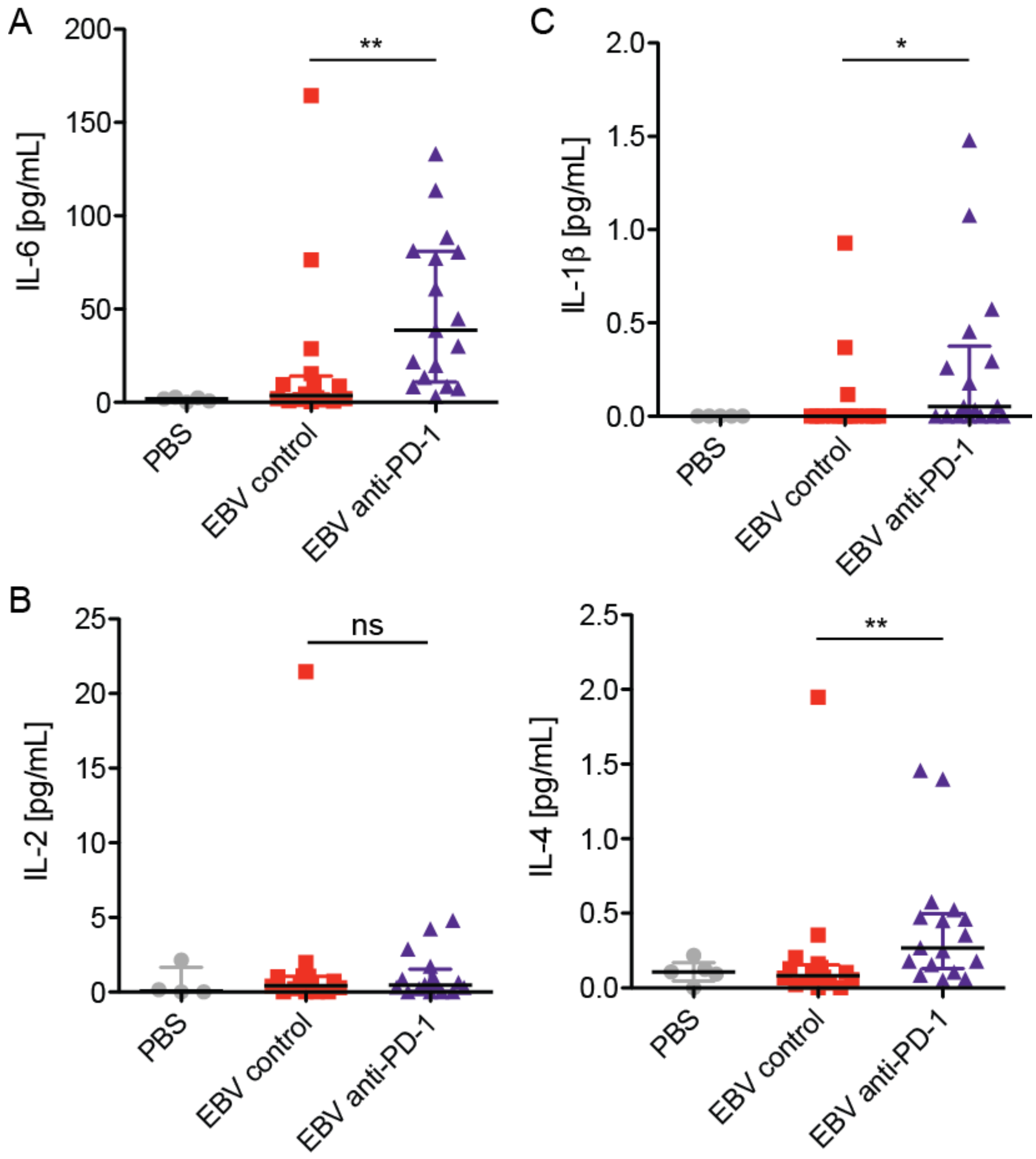

Figure S8: Treatment with anti-PD-1 antibodies results in higher levels of proinflammatory cytokines. A-C) Serum cytokines at the time of sacrifice. Data were analyzed using the Kruskal-Wallis test (IL-6: p=0.0004, IL-2: p=0.5890, IL-1 β : p=0.0317, IL-4: p=0.0106), and statistics from the Dunn's post-test are displayed. In all panels, data displayed were combined from 3 independent experiments, with 5-17 animals per group in total. Each point represents one animal. Data are shown as the median and interquartile range. *, p<0.05, **, p<0.01, ns = not significant.
