# Supplementary material for: CD8+ T cells retain protective functions despite sustained inhibitory receptor expression during Epstein-Barr virus infection in vivo
Source: PLoS Pathog. 2019 May 30;15(5):e1007748. doi: 10.1371/journal.ppat.1007748 (PMC6542544; doi:10.1371/journal.ppat.1007748)
Supplement: S5 Fig — A) Microarray data from Fig 3 examining genes found in the GO term for T cell mediated cytotoxicity (GO:0001913). Data are separated by species. B) Microarray data from Fig 3 examining genes found in the GO term for T cell costimulation (GO:0031295), separated by species. (PDF) [file ppat.1007748.s005.pdf]

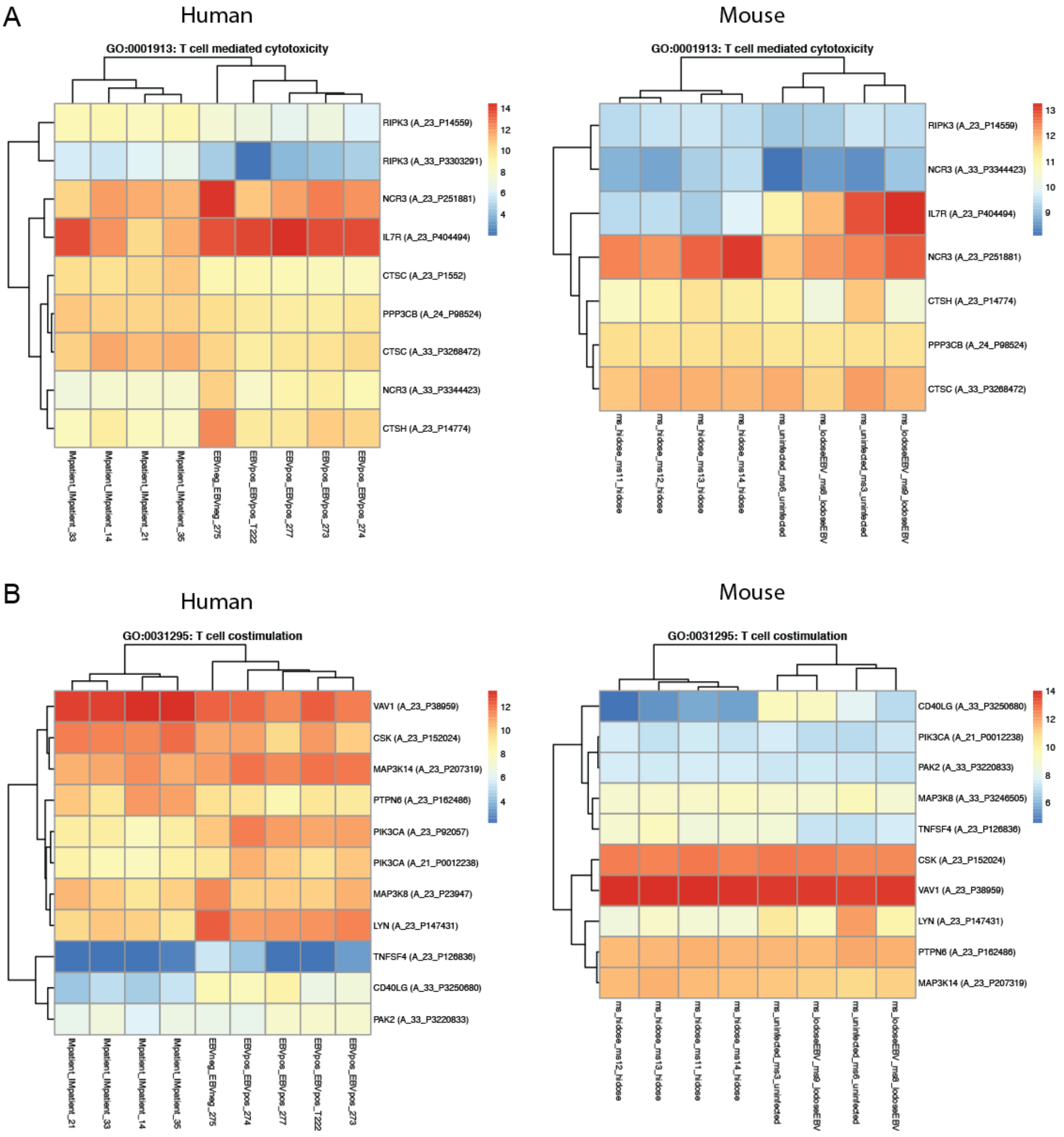

Figure S5: IM patients and huNSG mice infected with EBV retain unique transcriptional characteristics. A) Microarray data from Figure 3 examining genes found in the GO term for T cell mediated cytotoxicity (GO:0001913). Data are separated by species. **B)** Microarray data from Figure 3 examining genes found in the GO term for T cell costimulation (GO:0031295), separated by species.
